# Real Way to Target Gram-Negative Pathogens: Discovery of a Novel Helicobacter pylori Antibiotic Class

**DOI:** 10.1021/acs.jmedchem.5c00112

**Published:** 2025-03-31

**Authors:** Jonah Pascal Propp, Damien Oz Castor, M. Ashley Spies

**Affiliations:** † Department of Pharmaceutical Sciences and Experimental Therapeutics, 15509 The University of Iowa College of Pharmacy, Iowa City, Iowa 52242, United States; ‡ Department of Biochemistry, Carver College of Medicine, The University of Iowa Roy J and Lucille A Carver College of Medicine, Iowa City, Iowa 52242-1109, United States

## Abstract

In an era of escalating antibiotic resistance, there is a pressing need for innovative strategies to develop novel antibiotics. Gram-negative bacteria, characterized by their robust dual-membrane, are intrinsically resistant to a wide range of antibiotics and can readily develop new resistances. Members of this bacterial class comprise numerous pathogenic organisms, including the primary cause of gastric cancer, Helicobacter pylori. In this study, we used the Giga-sized collection of theoretical molecules inside Enamine’s REAL Space to identify inhibitors for H. pylori glutamate racemase. These compounds displayed a diverse range of activity in preventing H. pylori growth, with our most potent hits capable of selective full growth inhibition for metronidazole and clarithromycin resistant H. pylori strains. Alongside the introduction of a novel antibiotic class for this carcinogenic pathogen, our unique implementation of REAL Space holds great promise for Gram-negative antibiotic development as a whole.

## Graphical abstract

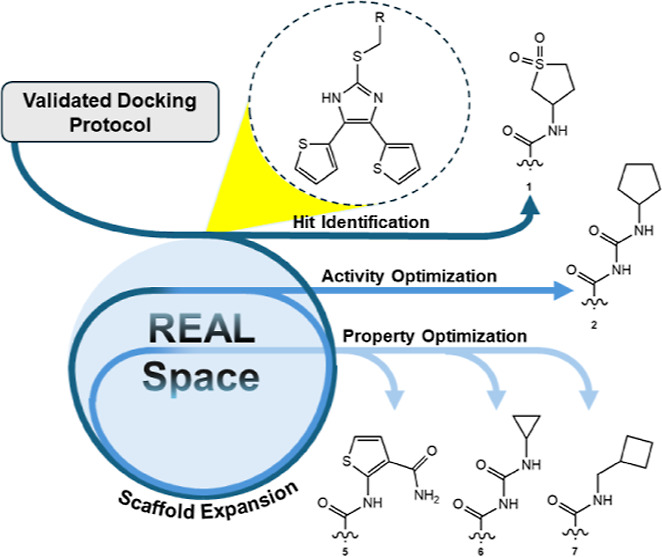

## Introduction

### Helicobacter pylori: A Clinically Relevant Pathogen

More than half of all humans are infected with the gastrointestinal pathogen H. pylori.^1^ This Gram-negative bacterium is the primary cause of gastric cancer, the fifth most common and third deadliest cancer worldwide.^2,3^ Additionally, those infected with H. pylori are at greater risk for gastritis and peptic ulcers.^4^ The current standard of care requires at least two nonselective antibiotics, often clarithromycin and metronidazole, causing widespread disruption of the gut microbiome.^5^ These conventional therapies are under threat as 30% of H. pylori strains show resistance to a commonly prescribed antibiotic, and 26% show double resistance.^6^ The lack of species-specific therapies combined with growing antibiotic resistance presents an urgent need for innovative antibiotic research to combat the pervasive health challenges posed by H. pylori infections.^4^

Developing antibiotics that target H. pylori is particularly difficult due to the inherent complexities associated with Gram-negative bacteria.^7^ Briefly, Gram-negative bacteria are characterized by their cellular envelope, with the peptidoglycan layer encapsulated by an inner and outer membrane. The outer membrane generally restricts entry for large hydrophobic molecules, while the inner membrane restricts entry for hydrophilic ones.^8^ Coupled with efflux pumps to remove penetrating compounds, Gram-negative cell envelopes present a robust barrier rendering species such as H. pylori resistant to a wide range of antimicrobial agents.^9^ For compounds to navigate this barrier, they must possess favorable physicochemical properties that enable transit through the outer membrane’s narrow porin channels while evading expulsion by efflux pumps.^10^ These properties include molecular size, globularity, and a balance between hydrophilicity and hydrophobicity, but the optimal combination is both species and chemotype-speicifc.^10^ Therefore, the design of novel antibiotics for H. pylori and other Gram-negative bacteria necessitates a meticulous and strategic approach, focused on exploring different molecular properties to ensure effective penetration and retention within the bacterial cell.

### Failure of the Current Discovery Paradigm

Faced with this formidable bacterial cell envelope, industrial drug discovery campaigns have been plagued by low success rates in screening efforts against Gram-negative pathogens.^11^ To address the global rise in antibiotic resistance, research groups have largely turned to modifying existing therapeutic pharmacophores, resulting in a lack of structural diversity among antibiotics.^12^ As of 2021, only a quarter of all antibiotics in clinical development represented a novel structural class, and just four targeted Gram-negative pathogens.^13^ This lack of innovation presents inherent challenges as bacteria become increasingly less susceptible to commonly prescribed antibiotic classes.^14^ Consequently, there is a pressing need to rethink the drug discovery process with a focus on structural novelty and diversity to effectively target Gram-negative pathogens such as H. pylori.

### Leveraging Enamine REAL Space

Enamine’s REadily AccessibLe (REAL) Space has emerged as a transformative resource to overcome various obstacles in the drug discovery process.^15,16^ REAL Space currently boasts over 50 billion drug-like small molecules, rapidly synthesized from building blocks and predefined reactions.^17^ Our group previously utilized REAL Space to identify potent disruptors of the RAD52-ssDNA interaction, providing novel anticancer drug leads.^15^ Alongside the unparalleled access to new compositions of matter, using REAL space allows for hit enumeration by targeted in silico screening of REAL analogs.^18^ This approach allows one to fine-tune physicochemical properties for enhanced Gram-negative penetration, accelerating the process from initial discovery to an optimized lead. However, the promise of Giga-sized chemical space can only be realized when a suitable target for in silico screening is identified.

### HpMurI as a Drug Target

Glutamate racemase (MurI) is an essential bacterial enzyme, responsible for catalyzing the stereoinversion of glutamate.^19^ d-glutamate is integrated in the pentapeptide side chain that bridges adjacent glycan polymers, reinforcing the peptidoglycan layer and safeguarding bacterial cells against osmotic rupture.^19^ Targeting enzymes involved with peptidoglycan synthesis has proved to be highly successful in antibiotic discovery.^20^ In the quest to develop a species-specific therapeutic for H. pylori, a team at AstraZeneca performed a high-throughput screening campaign targeting H. pylori MurI (HpMurI).^21^ HpMurI and other cofactor independent racemases perform catalysis through a unique mechanism, employing a cysteine dyad as an alternating acid/base pair.^22^ To accomplish stereoinversion, MurI must alter the electrostatic environment surrounding glutamate to allow for deprotonation of the poorly acidic Cα proton.^23^ This conserved mechanism and complex chemistry make the active site a very poor target for species-specific inhibition. By exploiting the structural differences of MurI across bacteria species, the AstraZeneca team identified two allosteric scaffolds selective for HpMurI.^21^

The first allosteric inhibitor published, designated as “compound **A**”, was found to bind to a novel cryptic pocket via X-ray crystallography (Figure 1a, red).^21^ Despite optimization efforts, compound **A** did not reach clinical candidacy due to poor solubility and high plasma protein binding (PPB).^24^ Previous work in our lab to uncover an allosteric mechanism has shown that compound **A** disrupts catalysis by dampening the enzyme’s flexibility.^25^ This finding was a departure from the previously proposed hypothesis that compound **A** inhibited HpMurI via an uncompetitive mechanism.^21^ Our research also identified a coupled motion network across the HpMurI homodimer that became severed upon cryptic allosteric inhibition.^26^ In characterizing these hallmarks of allostery, we also discovered a collection of natural-product inhibitors targeted at the cryptic pocket.^26^ These studies have both served to validate HpMurI as a drug target, while demonstrating the obstacles in optimizing physicochemical properties for antibiotic development.

**Figure 1:**
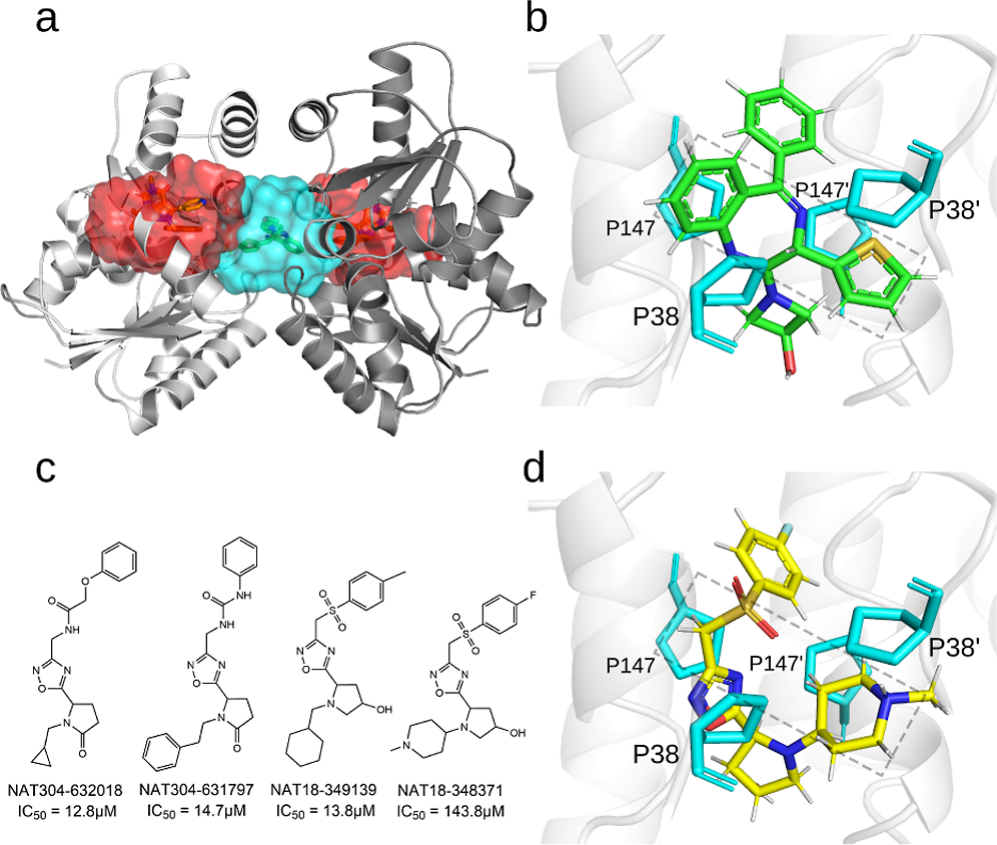
Structure of the HpMurI homodimer and allosteric inhibitors. (a) Hybrid crystal structures of the HpMurI head-to-head homodimer with allosteric pockets highlighted in red for the cryptic pockets (PDB: 2JFZ) and turquoise for the dimer interface (PDB: 2W4I). The two monomers are distinguished by different shades of gray. (b) Crystal pose of compound **B** bound in the HpMurI dimer interface, with the four proline residues comprising the proline groove highlighted in turquoise. The two prolines from monomer A are distinguished by an apostrophe. (c) Lewis structure of four inhibitors identified from the AnalytiCon NATx collection. AnalytiCon ID and IC_50_ values against HpMurI are listed. (d) Docking pose of NAT18–348371, a representative hit from the NATx library.

The second series of allosteric inhibitors discovered by the AstraZeneca team were found to inhibit HpMurI via binding at the dimer interface (Figure 1a, turquoise and Figure 1b).^27^ Like the first series, these compounds suffered from high PPB, but extensive optimization resulted in viable leads for in vivo studies. Based on preliminary single-dose pharmacokinetics, the maximum dose regiment was expected to provide compound exposure levels above the minimum inhibitory concentration (MIC) for the entire experiment. However, the plasma concentration of the inhibitors dropped over the duration of the study and were above the MIC only ∼50% of the time. This was far below the >95% level presumed necessary for in vivo efficacy based on amoxicillin.^28^ This drop in plasma concentration for the inhibitors was hypothesized to result from the upregulation of the metabolic cytochrome P450 enzymes, leading the AstraZeneca team to abandon this series as well.^28^ Consequently, HpMurI remains a validated drug target with two known allosteric scaffolds, but no inhibitors in clinical development.

Guided by the structural data published by the team at AstraZeneca, we sought to identify potential H. pylori antibiotics by targeting the HpMurI dimer interface. Starting with decoy ligands, we used the Receiver Operating Characteristic (ROC) statistical method to optimize molecular docking for the dimer interface.^29^ We then validated this docking protocol by screening a library from AnalytiCon Discovery, GmbH (Potsdam), providing a small collection of HpMurI inhibitors. We next applied our virtual screen to Enamines REAL Space and found a structurally related hit. However, this compound was incapable of preventing H. pylori growth, likely due to unfavorable Gram-negative penetration. We therefore looked to expand upon this hit by screening chemically similar compounds from REAL Space and testing ligands with different properties. Doing so revealed a fully bacteriostatic compound sharing the same core scaffold as our first-generation REAL hit. This second-generation inhibitor possessed activity on par with H. pylori antimicrobials in clinical development,^28,30^ highlighting the ability to jump from a compound lacking an MIC to one capable of Gram-negative penetration and inhibition. Imaging of H. pylori cells treated with this inhibitor revealed altered cell wall structure and a loss of helical morphology. This supports not only antibacterial activity, but a possible role in reducing pathogenicity in H. pylori and sub-MIC concentrations.

A final round of optimization inside REAL Space provided additional scaffolds with comparable activity, all of which showed selective growth inhibition with no impact on three common gut bacteria species. These analogs also possessed diverse estimates for PPB, with many showing large improvements over leads identified by the AstraZeneca team. Alongside our introduction of a novel antibiotic class for the pathogen driving the second leading cause of cancer-related deaths,^31^ iterative rounds of optimization inside REAL Space possess far reaching potential for drug development targeting Gram-negative bacteria as a whole.

## Results

### ROCs against the Dimer Interface

Previously, our lab identified natural product inhibitors that target the HpMurI cryptic allosteric pocket by employing ROC analysis to evaluate docking protocols.^26^ In this study, we applied the same logic to the dimer interface, using ROC analysis to select an optimal system for docking into the crystal structure of the AstraZeneca team’s inhibitor (compound **B**) bound at this site. Briefly, ROC analysis is a way to characterize a virtual screen’s ability to discriminate between known actives and assumed inactives (i.e., hypothetical decoys in this case) by plotting the true positive rate (sensitivity) against the false positive rate (1-specificity) at various score thresholds. The area under the ROC curve (AUC) quantifies the overall predictive power of a docking protocol, providing a single metric to compare different docking methods. A perfect virtual screen would have an AUC of 1, while an AUC of 0.5 corresponds to random chance.

Early comparisons of docking performance between different docking approaches and scoring functions led us to select the molecular operating environment (MOE) (chemical computing group) for all protocols discussed. Initial tests were performed using three active compounds reported by the AstraZeneca team, and 300 decoys from the Directory of Useful Decoys enhanced (DUD-E).^32^ These decoys share similar physicochemical properties to the actives, but are structurally unique and therefore presumed to be biologically inactive. Using the DUD-E ligands allowed for rapid characterization of docking accuracy, with only the known actives available.

This process led us to use the ASE scoring function in MOE, which provided an AUC of 0.9010 (Figure S1a). To validate our virtual screening method, we applied it to a previously purchased AnalytiCon library of 18,565 compounds (NATx). We selected 65 compounds for screening via differential scanning fluorimetry (DSF) and enzyme activity assays. Interestingly, none of the selected ligands appeared as hits and the docking parameters were reexamined with the addition of these 65 true negatives.

After incorporating ligands that had erroneously appeared as hits from the top docking protocol, we found that the GBVI/WSA scoring function performed better at distinguishing true positives from true negatives (Figure S1b,c). Performing an energy minimization in MOE increased the false positive rate, while just using the protonate3D^33^ utility to reassign residue protonation states increased the AUC to 0.9893. Using these improved parameters, the NATx library was screened again for potential inhibitors. Compound **B** and other dimer interface inhibitors from AstraZeneca provided docking scores of −9.3 to −10.1 kcal/mol, therefore, a cutoff of −10.0 kcal/mol was applied to determine “hit compounds” via docking.

### AnalytiCon Hits

Twelve compounds were cherry-picked from the AnalytiCon NATx Library, and four structurally related scaffolds were found to be inhibitors of HpMurI (Figure 1c). These compounds all share a 2,4-diazafuran center, connected to two rings at the 5 position and a linker to an aromatic ring at the 3 position (IUPAC numbering). In comparing the docked pose of these ligands to the crystal structure of compound **B**, a critical observation was made regarding the placement of aromatic rings. Compound **B** contains two aromatic rings flanked by mirroring proline residues (Pro38 and Pro147) from both monomers, what we will be referring to as the “proline groove” (Figure 1b). Highlighted with a rectangle in Figure 1b, these diagonal aromatic rings are in fact shared among all dimer interface inhibitors identified by the AstraZeneca team.

In contrast, the compounds we identified from the AnalytiCon library did not align aromatic groups within this proline groove based on their docking poses. Instead, each placed a phenyl ring at the entryway to the dimer interface with the 2,4-diazafuran moiety pushed outside the box, as shown in Figure 1d. Furthermore, these hits exhibited greater conformational freedom compared to the fused ring system of AstraZeneca’s compounds. We suspected that these differences in molecular architecture were influencing our inhibitor’s weaker activity, and thus sought to explore the structure–activity relationship (SAR) and larger chemical space via focused screening of Enamine’s REAL Space.

### Jump into Enamine’s REAL Space

With our docking protocol now validated through the identification of hits in the AnalytiCon NATx library, we developed a hierarchical screening platform targeting HpMurI. A representative sampling of 833k molecules from REAL Space were filtered according to 2–5 aromatic groups, MW300-450, and a log *P* less than 4. The decision to select for multiaromatic containing compounds with restricted size was aimed at enriching fused aromatic scaffolds. As outlined in Figure 1b,d, we hypothesized that greater complement to the proline groove through multiring systems like compound **B** would result in improved compound activity.

The resulting library of 81 k was docked into the HpMurI dimer interface, and four compounds were ordered based on favorable docking scores and placement of one or more aromatic groups in the proline groove. Only one ligand, designated compound **1** (**1**), displayed inhibition with an IC_50_ of 42.5 μM (Figure S2). The docking pose and ligand interaction map for compound **1** are shown in Figure 2a,b.

**Figure 2:**
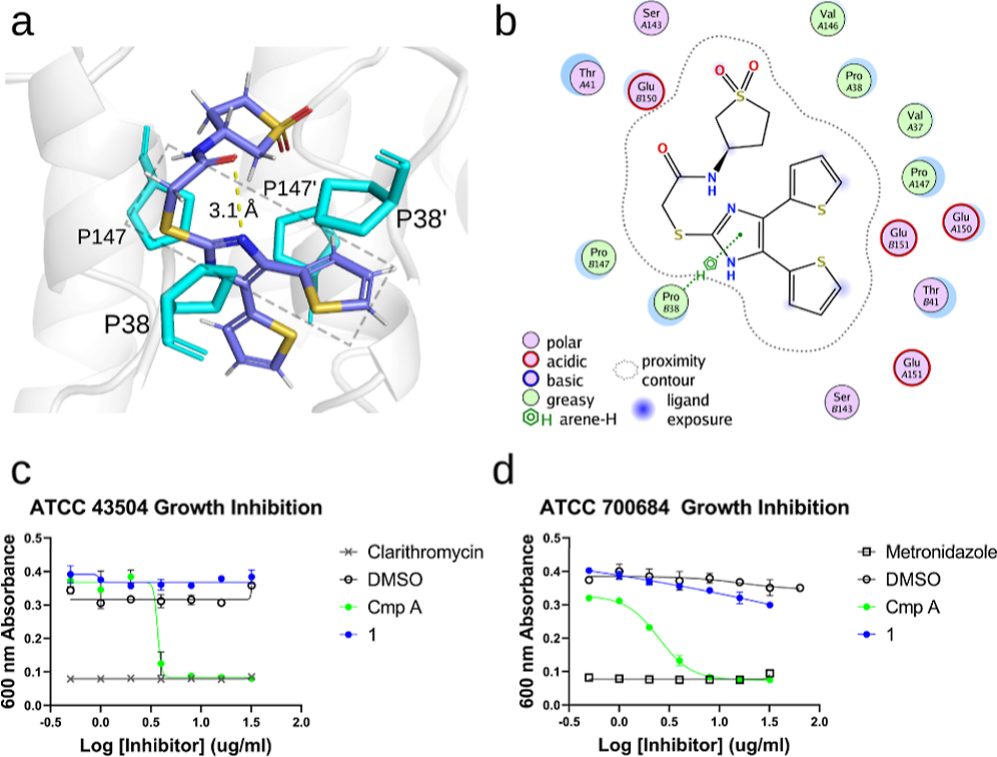
Enamine REAL hit shows greater compliment to the proline groove. (a) Pose of compound **1** docked into the HpMurI dimer interface, with proline groove residues highlighted in turquoise (PDBID: 2W4I used for modeling). The overlaid box depicts the region of the proline groove that is occupied by two aromatic rings in the compound **B**-bound crystal structure. The docked pose of **1** places one aromatic group, a thiophene ring, in the same area as compound **B**’s thiophene. The other side of the proline groove is filled by the linker region of **1**. (b) Ligand interaction map of **1** bound in the dimer interface generated in MOE. (c,d) Antibacterial activity of **1** and controls against H. pylori strains ATCC 43504 (metronidazole resistant) and ATCC 700684 (clarithromycin resistant).

One of the thiophene rings occupies the right half of the proline groove, while the left half is filled by the sulfide/amide linker of **1**. The orientation of the amide carbonyl allows for intramolecular stabilization with the nearby imidazole nitrogen. The thiophene placement resembles Compound **B**’s thiophene, only rotated 180 deg. Remarkably, the binding pose and Lewis structure of **1** show great similarity with the initial AnalytiCon NATx hits (Figure S3a,b). While unintentional, this finding bolstered our confidence in the Enamine REAL hit and characterizes our computational screen as optimizing a novel pharmacophore for the HpMurI dimer interface.

To examine the effect of **1** on H. pylori cell cultures, we performed a growth inhibition assay using two antibiotic resistant H. pylori strains (Figure 2c,d). For controls, we used the standard of care antibiotics clarithromycin and metronidazole to define full inhibition (ATCC 43504 was metronidazole resistant but clarithromycin susceptible and ATCC 700684 was clarithromycin resistant but metronidazole susceptible), along with AstraZeneca’s compound **A**. Compound **A** possessed an MIC of 8 μg/mL against both strains, relatively close to the value of 4 μg/mL published by the AstraZeneca team.^21^ Unfortunately, **1** showed no ability to inhibit H. pylori growth for ATCC 43504 (Figure 2c), and showed only 13% inhibition for ATCC 700684 (Figure 2d) at the highest concentration tested. We hypothesized that this was due to poor uptake and/or rapid efflux by the bacteria. To overcome this lack of biological activity, we jumped back into REAL Space with **1** anchoring our screen to an active chemotype.

### First REAL Optimization Screen

Using the program infiniSee^35^ (BioSolvIT) to navigate REAL Space, we selected 500 compounds with >90% similarity to **1** for docking using our optimized protocol. All top ranked ligands had the same 4,5-dithienylimidazole core and were visually inspected to select a focused analog series to explore variations in the amide linker, aliphatic ring region, and Log *P* (Table 1). Three analogs were chosen for synthesis, and upon delivery were screened via enzymatic activity assays and DSF.

**Table 1:**
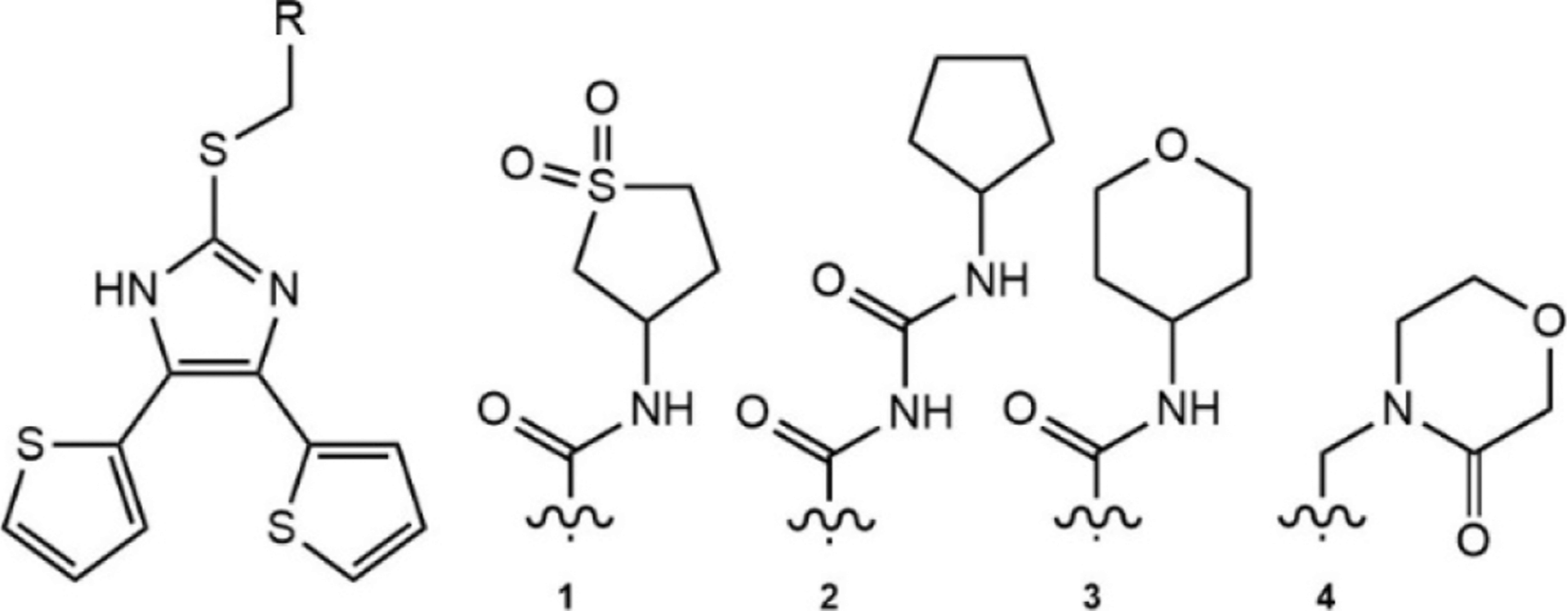
Structure of 4,5-Dithienylimidazole Ligands and Associated Values

compound	thermal Shift (°C)^a^	IC_50_ ^b^ (μM)	Log PI^c^
**1**	0.7	42.5 ± 6.7	2.95
**2**	1.6	2.0 ± 0.5	3.87
**3**	0.5	41.7 ± 1.6	3.54
**4**	0.2	69.3 ± 8.1	3.12
**A**	3.8	5.9 ± 0.6	3.73

a HpMurI unfolding stabilization determined from DSF at 250 μM ligand concentration.

b Coupled enzymatic activity measuring HpMurI mediated turnover of d-glutamate to l-glutamate. Regression error is reported based on the 95% confidence interval of curve fitting in GraphPad Prism.

c Log octanol/water coefficient, reported as the consensus of various lipophilicity measurements from SwissADME.^34^

Compound **2** inhibited HpMurI with an IC_50_ of 2 μM, making it more potent than compound **A** in our activity assay (Figure S4a). Compound **3** was the only ligand that shared the same linker as **1** and had a near identical IC_50_ of 41.7 μM. Compound **4** contained a shortened linker with no amide group and had an IC_50_ of 69.3 μM. This trend in activity was mirrored in the stabilization of HpMurI seen from DSF. The weakest inhibitor, **4**, showed minimal deviation from the DMSO control. **1** and **3** gave 0.5–0.7 °C of stabilization, while **2** gave 1.6 °C of stabilization. The correlation between greater HpMurI stabilization from DSF and lower IC_50_ values indicate that our inhibitors may operate through reducing enzyme flexibility, as we have shown for compound **A**.^25^ With a focused set of analogs based on **1** exhibiting varied activity and Log *P* (Table 1), we returned to examining H. pylori growth inhibition.

### Antibiotic Activity of the REAL Analogs

Each of the second-generation inhibitors showed improved antibacterial activity toward the two antibiotic resistant strains of H. pylori (Table 2, and Figure S5a,b). However, only the most potent and hydrophobic inhibitor, **2**, was capable of fully preventing H. pylori growth. Compounds **3** and **4** still displayed improved growth inhibition compared to **1** (Figure S5 and Table S1) and were capable of fully inhibiting ATCC 700392 (no antibiotic resistance) at the highest concentration tested. From this, we interpreted that raising Log *P* improved H. pylori growth inhibition.

**Table 2:** MIC Values of HpMurI Inhibitors

cmp	43504 MIC (μg/mL)	700392 MIC (μg/mL)	700684 MIC (μg/mL)
**1**	>64	>64	>64
**2**	16	16	16
**3**	>64	64	>64
**4**	>64	64	>64
**A**	8	8	8
clarithromycin	<0.25	<0.25	>64
metronidazole	32	4	<0.25

### Loss of Antibiotic Activity with Excess d-Glutamate

To provide additional evidence for HpMurI being the antibacterial target of our inhibitors, we performed modified MIC experiments in the presence of 1 mM d-glutamate. While one would expect poor bacterial penetration from d-glutamate, H. pylori has a well characterized glutamate transporter.^36^ Guided by previous work, we developed a modified MIC assay to examine the impact of extracellular d-glutamate on H. pylori growth inhibition.

Compound **2** and compound **A** both show reduced antibacterial activity in the presence of d-glutamate (Figure S6). In contrast, metronidazole showed full growth inhibition at 32 μg/mL weather or not d-glutamate was present. The loss of antibacterial activity for the HpMurI targeting compounds suggests that they disrupt proper d-glutamate production, which is solely produced by HpMurI.^19^

### Scanning Electron Microscopy

We next sought to evaluate how **2** impacts H. pylori morphology. Liquid cultures of H. pylori exposed to varying concentration of **2** were incubated for 72 h and mounted for Scanning Electron Microscopy (SEM). Each sample showed H. pylori in helical, rod, and coccoid forms to differing degrees. The antibiotic-free sample (Figure 3a) showed the most classical representation of H. pylori, with most in the helical shape associated with pathogenicity.^37^ We observed altered H. pylori cell envelope structure when treated with sub-MIC concentrations of **2**, with the appearance of small ruptures at 2 μg/mL (Figure 3b), and reduced helical populations at 4 μg/mL (Figure 3c). The samples treated with the highest concentration of **2** appeared to both lose helical structure and have an altered cell wall surface (Figure 3d).

**Figure 3:**
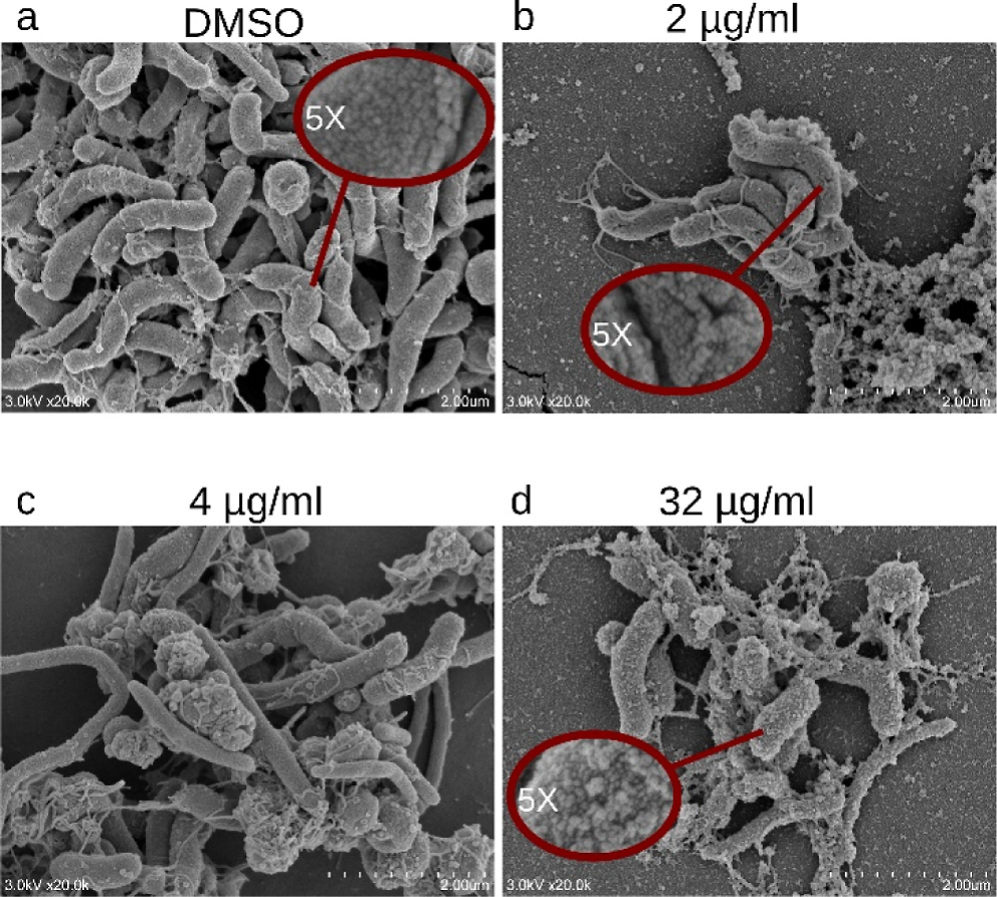
Inhibitor 2 Disrupts H. pylori Morphology. (a–d) SEM images of H. pylori cultures incubated with increasing concentrations of compound **2**. We observe a loss of helical structure and a loss of smooth cellular surface with higher concentrations of **2**.

### Second REAL Optimization Screen

With visual evidence of the activity of **2**, and a better grasp of how ligand structure and Log *P* impact activity, a second round of inhibitor optimization was explored using **2** as the reference molecule for infiniSee. Following docking, 16 compounds with greater structural diversity were selected for custom synthesis. These largely focused on substituting the linker and adjacent ring region, as this was the most common derivatization within highly similar REAL Space. Ten of these contained the 4,5-dithienylimidazole core, with all but two showing activity against HpMurI (Table S1) While none of the ligands showed improved growth inhibition, several had IC_50_ and MIC values equivalent to **2** (Table 3). In general, replacement of the 4,5-dithienylimidazole core abolished activity. Other poorly tolerated changes included lengthening the linker of **1** by a carbene group, and addition of a methyl substituent in the linker region. For a full overview of chemical structures and activity data, refer to Table S1.

**Table 3:**
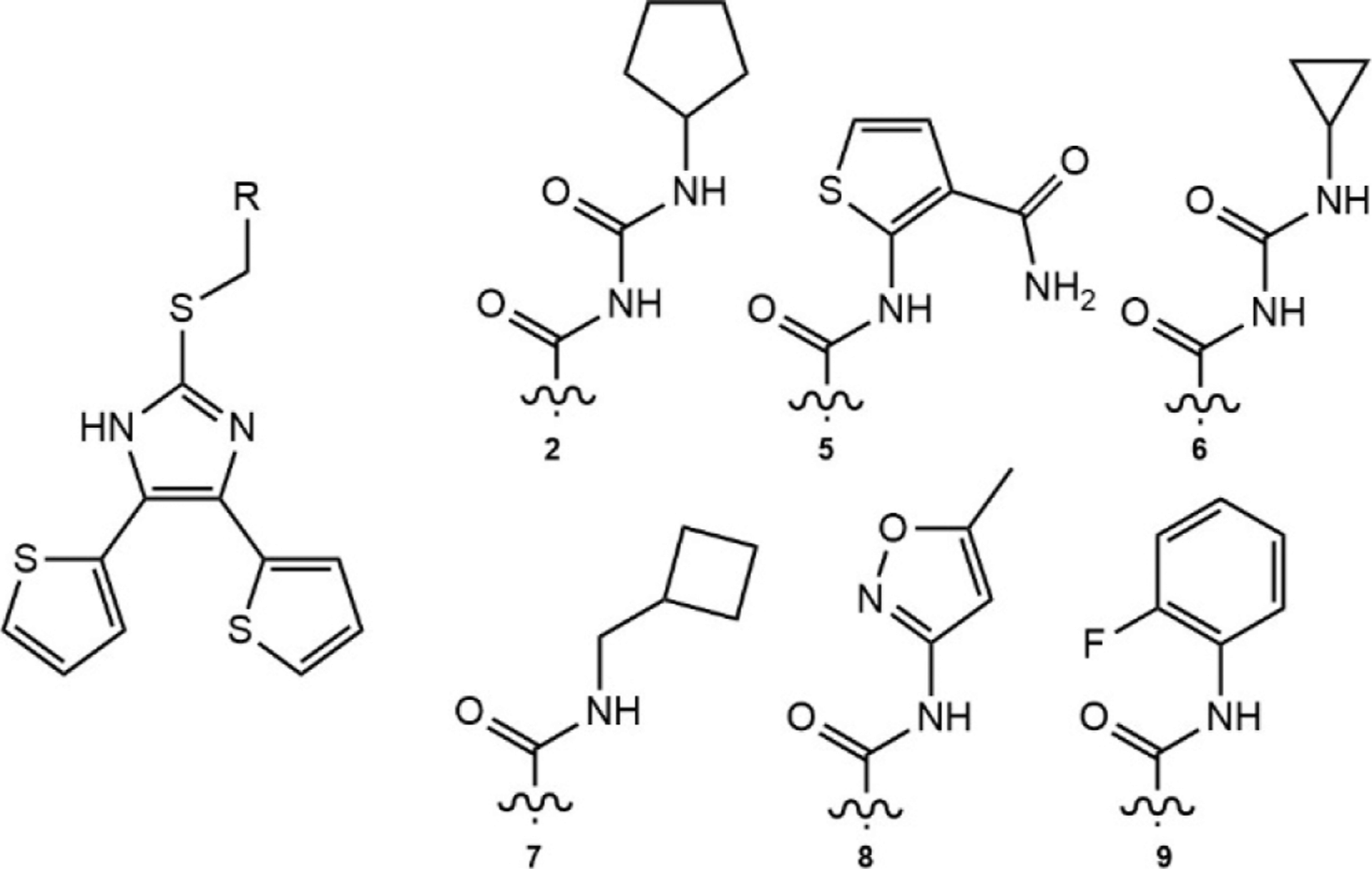
Final Screen of REAL Space Uncovers Additional Active Scaffolds^a^

cmp	43504 MIC	700392 MIC	IC_50_ ^b^ (μM)	Log *P*	PPB (%)^b^
**2**	16	16	2.0 ± 0.5	3.87	90.5
**5**	32	32	1.6 ± 0.3	3.76	93.7
**6**	32	32	16.8 ± 4.0	3.21	84.9
**7**	32	16	15.6 ± 1.4	3.50	93.5
**8**	16	16	8.7 ± 1.9	3.50	91.7
**9**	16	16	10.7 ± 1.7	4.72	98.1
**A**	8	8	5.9 ± 0.6	3.73	97.0
**B**	8^c^	N.D.	0.5^c^	3.42	96.4

a IC50 values along with regression error from the 95% confidence interval of the curve fit are reported.

b PPB estimate created by taking the consensus value from three different PPB prediction models.

c Value taken from Geng et al.^27^

### Species Specificity

To evaluate antibiotic specificity, the six most potent H. pylori inhibitors were tested against three common gut bacterial strains: E. coli MG1655,^38^ E. Faecalis OG1RF,^39^ and B. Subtilis PY79.^40^ The compounds were administered at concentrations ranging from 0.5 to 64 μg/mL. Notably, the REAL inhibitors exhibited minimal deviation from the DMSO control across all experiments and did not demonstrate any discernible MIC values (Table 4 and Figure S7). While the scope of this preliminary investigation was limited, it offers valuable insight into antibacterial selectivity, suggesting a favorable safety profile in terms of microbiome disruption. These results mirror AstraZeneca’s selectivity profile for compound **B** *,* which showed high selectivity for H. pylori *.* Collectively, these findings unveil a novel class of H. pylori antibiotics, all characterized by a central 4,5-dithienylimidazole functionality.

**Table 4:** HpMurI Inhibitors Display Bacterial Selectivity

compound	E. coli (μg/mL)	E. Faecalis (μg/mL)	B. Subtilis (μg/mL)
**2**	>64	>64	>64
**5**	>64	>64	>64
**6**	>64	>64	>64
**7**	>64	>64	>64
**8**	>64	>64	>64
**9**	>64	>64	>64
ampicillin	32	4	>64
clarithromycin	>64	4	<0.5

## Discussion

This work reveals a promising approach to discovering effective and selective therapeutics against Gram-negative bacteria such as H. pylori. In doing so, we share a new class of antibiotics which inhibit HpMurI, a coveted target in antibiotic development.^27^ As our ligands are predicted to show allosteric binding, they contribute to the growing promise of allostery in structure based drug discovery. While allostery holds the potential to reveal new druggable pockets with improved specificity, only 19 FDA approved drugs are allosteric in nature, and just one was identified via computational-based methods.^41^ Our work underscores the importance of combining computational tools with experimental validation to expand the landscape of allosteric drug discovery.

By integrating the structural knowledge gained from previous AstraZeneca inhibitors with virtual screening techniques validated by ROC analysis and the NATx library, we identified a hit inside Enamine’s REAL Space. After undergoing a focused optimization screen inside REAL Space, we identified **2**, which displayed vastly improved activity against H. pylori cells. A final round of optimization using REAL Space produced five compounds with comparable activity to **2** and varied estimated PPB. These all showed vast superiority to our initial hit, **1**, which failed to inhibit H. pylori growth. For reference, a diagram of the project workflow is presented in Figure 4a. Although we could not have anticipated which changes would result in growth inhibition, our work demonstrates that minor structural alterations can lead to improved Gram-negative penetration. This highlights the importance of iterative docking using curated chemical space to navigate the complex cell envelope of Gram-negative pathogens.

**Figure 4:**
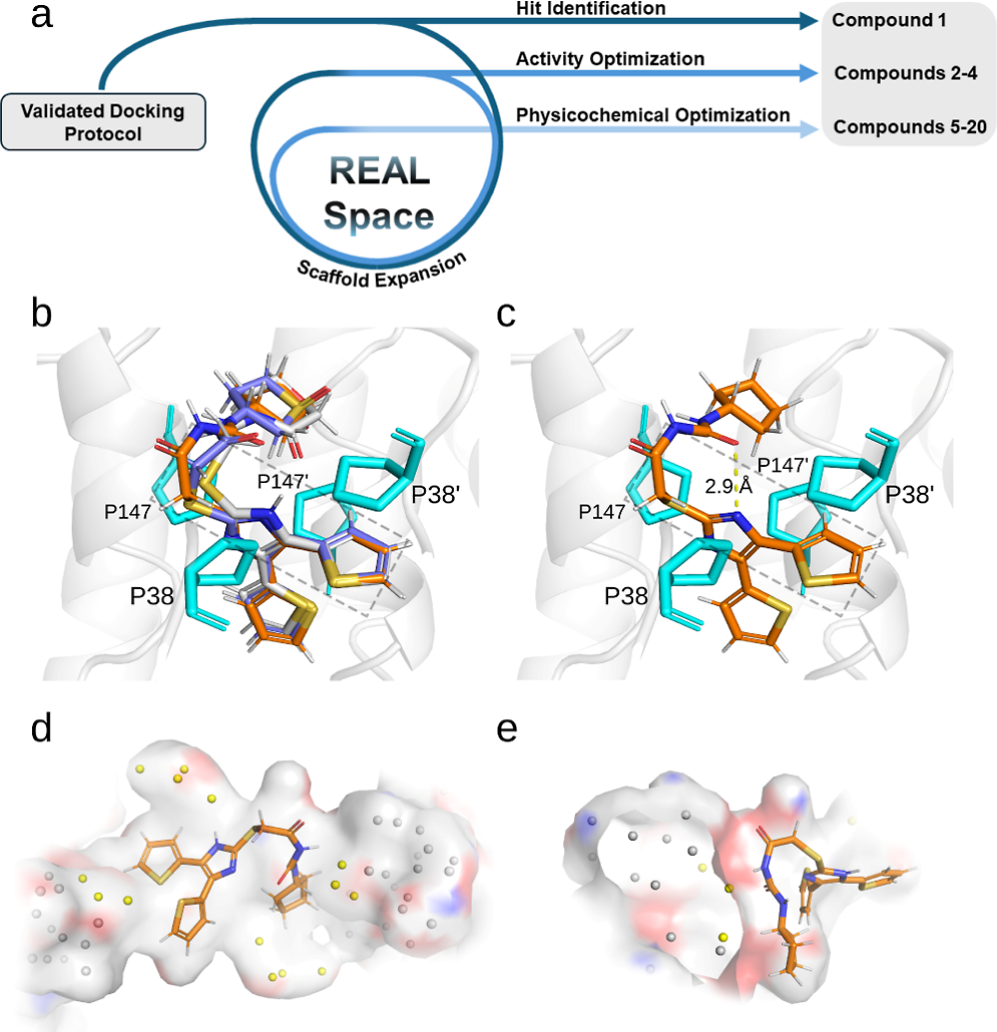
Improved inhibitors extend into the proline groove. (a) Schematic of project workflow. After using ROC analysis and the AnalytiCon NATx library to validate docking, we identified a hit inside REAL Space. We then underwent scaffold expansion inside REAL Space to improve compound activity, providing compounds **2–4**. A final round of optimization yielded several inhibitors with more favorable physicochemical properties. (b) Overlay of compounds **1** (indigo), **2** (orange), and **4** (white) docked into the HpMurI dimer interface (PDBID: 2W4I used for modeling). The box surrounding the proline groove exemplifies how increased occupancy correlates with ligand potency. Inhibitor **3** has been omitted for clarity as it shows identical poses in the linker positioning at **1**. As we expand the linker length, we see greater filling of the left portion of the proline groove, and more potent HpMurI inhibition. (c) Docked pose of **1** with intramolecular contact designated with a dashed yellow line. (d) Surface rendering of the HpMurI dimer interface with **2** and water molecules from 2W4I modeled in. The waters closest to the ligand are colored yellow, while those sitting in the pocket entryways are shown in gray. (e) View of the pocket entryway located closest to the linker region of **2**.

### ROCs

While decoys generated from the DUD-E server provided early probes for our docking protocols, the limitations of using theoretical negatives became apparent after screening 65 inactive ligands from the NATx library. After incorporating these true negatives, the ASE scoring function showed an expected reduction in the AUC. At the same time, applying the GBVI/WSA function to the true negatives showed superiority, and thus was chosen for virtual screening. This process highlights the advantages of known true negatives in optimizing molecular docking.

### Initial REAL Hit

Through library filtering and medium-scale docking, we were able to identify a pharmacophore better suited to the proline groove than the AnalytiCon hits. Compound **1** satisfied two of our primary structural goals, showing decreased flexibility with a more rigid ring system and an aromatic sitting in half of the proline groove. Early in the optimization of docking protocols, we explored alternative means to target this pocket through the use of induced fit, a manually defined pharmacophore, and crystal electron density (Figure S8). In all cases except the pharmacophore method, these gave worse AUC values compared to standard docking. In contrast, the use of a pharmacophore based on compound **B** in the 2W4I crystal structure (specifically containing two aromatic groups in the proline groove) artificially inflated the AUC, flagging only AstraZeneca’s ligands as hits. While appearing ideal, this protocol found no hits when applied to the AnalytiCon NATx library, indicating that the pharmacophore was too restrictive. Indeed, had we used the pharmacophore for screening REAL Space, we would have never identified inhibitor **1**. This is because while **1** places an aromatic in one side of the proline groove, the other side is filled by the amide linker (Figure 2a), and thus would be eliminated as a hit. The unbiased approach at filtering REAL Space based on simple, yet specific molecular properties yielded a reasonable number of compounds to dock with greater complement to the pocket.

### Compound SAR

Based on the docking poses and activity of the inhibitors discovered, we can make some inferences on SAR. To simplify this discussion, we will focus on the docked poses of **1–4**. Each 4,5-dithienylimidazole core and ring region remain relatively unchanged compared to the linker regions (Figure 4b). The weakest inhibitor (**4**, white) occupies the least volume in the pocket, while the best inhibitor (**2**, orange) occupies the most. This extra ligand volume stems from the increased size of the linker in **2**, which allows for direct hydrogen bonding to HpMurI (Figure S9c). When overlaid with compound **1**, we observe a clear correlation between increased occupancy of the proline groove, greater stabilization from DSF, and lower IC_50_ values. This is further exemplified by the near identical activities of compounds **1** and **3**, which share the same linker length and IC_50_ values. Notably, compounds with the same linker as **1** and **3**, but with aromatics in the ring region produced improved IC_50_ values between 1.7 and 10.7 μM (Table 3). Those with substituted aromatic rings showed the highest potency in terms of enzyme inhibition, but this did not consistently result in H. pylori growth inhibition. As **2** and **5** displayed a >20-fold improved IC_50_ over **1**, analogs with an extended linker or substituted aromatic ring region should be prioritized in future studies.

Compared to compound **A**, we observed partial inhibition of HpMurI for all the REAL compounds discovered, usually reaching ∼70% inhibition. This lack of full inhibition may be partially explained by the complexities arising from our reference/positive control, compound **A**. Compound **A** binds to a cryptic pocket near the C-terminal helix of both monomers in HpMurI, reducing the flexibility required for catalysis.^26^ Along with acting through a unique cryptic pocket with different binding stoichiometry, compound **A** suffers from poor solubility, possibly interfering with assay readout. Nevertheless, the similar maximum enzyme inhibition and full growth inhibition against H. pylori point toward a shared and effective antibacterial mechanism for the REAL compounds.

### Intramolecular Stabilization

A possible link between activity and structure lies in the intramolecular hydrogen bond formed between the amide linkers and the imidazole ring of the REAL inhibitors. As shown in Figures 2a and 4c, both **1** and **2** have carbonyl groups located within ∼3 Å from an imidazole nitrogen. Compound **2** possesses a larger linker, which repositions the carbonyl 0.3 Å closer to the imidazole nitrogen (Figure 4c). As this nitrogen is readily protonated, an intramolecular hydrogen bond may provide entropic stabilization of the docked pose of **2**, contributing to its increased potency. For comparison, the least active analog, **4**, contains no amide group in the linker. Instead, **4** has a carbonyl in the ring region, located at a much less favorable angle for hydrogen bonding (∼90° to the nitrogen–hydrogen bond). In summary, the linker region may play a dual role of filling the proline groove and stabilizing the bound conformation of ligands via intramolecular contact.

### Gram-Negative Penetration

In terms of antibacterial activity, we observe a trend likely dominated by hydrophobicity. Hydrophobicity is recognized as a crucial factor in Gram-negative permeation; however, no universal trend applies to all ligands and all species.^10^ In a 2020 review aggregating multiple studies of Gram-negative penetration for various compounds and species, contradictory findings were abundant.^10^ For example, sulfamoyladenosine accumulation in E. coli was positively correlated with increased hydrophobicity, whereas β-lactams demonstrated an inverse correlation with hydrophobicity. These data indicate that the optimal properties for Gram-negative penetration must be determined on a case-by-case basis, as different chemotypes show different trends against the same target species. In the case of our ligands, the possible influence of Log *P* in determining activity is supported by comparing compounds **1** and **3**. Despite the near identical docking poses and IC_50_ values of **1** and **3**, only **3** could reduce H. pylori growth. Compound **3** possessed a higher Log *P* (3.54 vs 2.95 for **1**), in alignment with the higher Log *P* seen for many of AstraZeneca’s inhibitors.^27^ The four compounds reaching an MIC of 16 μg/mL were **2**, **7**, **8**, and **9**, and had Log *P* values of 3.87, 4.20, 3.50, and 4.72, respectively. Only compound **6** shared the same extended linker as **2**, but showed reduced activity alongside a lower Log *P* of 3.21. With the exception of **6**, it appears that a Log *P* above 3.5 is needed for full growth inhibition of H. pylori within this pharmacophore. Conversely, some HpMurI inhibitors with a Log *P* above 3.5 failed to provide a MIC, as shown in Table S1. Therefore, there are likely multiple factors driving antibiotic activity, but increased hydrophobicity is the most readily apparent.

### Imaging of H pylori Treated with Compound **2**

When investigating morphological changes of H. pylori treated with **2**, we observe a clear reduction in helical populations at sub-MIC concentrations (Figure 3c) and altered cellular envelopes at various concentrations (Figure 3b,d). The loss of helical shape at sub-MIC concentrations may have great clinical relevance, as the helical structure of H. pylori is thought to be important for pathogencity.^42^ The changes in the cell surface mirror a recently published article detailing the impact of the natural product dioscin against H. pylori cells.^43^ In both cases, the once smooth surface develops a rougher texture, likely due to increased membrane vesicle secretion to improve bacterial survival. Curiously, we found elongated H. pylori cells at 4 μg/mL and above (Figure 3c,d), a possible side effect of disrupting peptidoglycan production. An increase in coccoid cells can be seen at 4 μg/mL, which is a known defensive tactic of H. pylori against antibiotics.^44^ Spigel et al. postulate that an ideal H. pylori antibiotic would avoid inducing coccoid formation,^43^ but we primarily observe these at sub-MIC concentrations. Taken together, our results clearly indicate a negative impact on cellular growth, and a potential to reduce pathogenicity at sub-MIC concentrations of inhibitor.

### H pylori Growth Rescue with d-Glutamate

To further validate that **2** targets HpMurI, we examined changes in MIC in growth media supplemented with 1 mM d-glutamate. Both compound **A** and **2** showed a 2-fold reduced MIC, indicating a clear connection between antibacterial activity and depletion of intracellular d-glutamate (Figure S6). The similar reduction in MIC, (32 μg/mL for **2** and 16 μg/mL for compound **A** when in the presence of d-glutamate) provides additional evidence of direct HpMurI engagement. This is further supported by the extensive validation performed by the AstraZeneca team. Along with a crystal structure of compound **A** bound to HpMurI, resistance mutations were generated for H. pylori, which identified several single amino acid mutations in the primary sequence of MurI. Taken in concert with inhibition data, DSF, bacterial selectivity, and morphological changes, we concluded that HpMurI is at the very least a primary target of compound **2**, as has been shown for compound **A**.

### Plasma Protein Binding Predictions

One barrier to AstraZeneca’s drug discovery campaign targeting HpMurI was the high PPB of their inhibitors. Despite extensive SAR on two allosteric scaffolds, their leads failed to reach plasma concentrations high enough for therapeutic efficacy. To provide an estimate of PPB, we used the web-based applications PreADMET,^45^ ADMElab,^46^ and admetSAR.^47^ We observed variations between prediction methods, but all ranked **6** as having significantly improved PPB (Table S2). To streamline this comparison, we report the average value across all three methods in Table 3. Aside from **9**, all top inhibitors showed lower PPB than compounds **A** and **B**, bolstering our confidence that these ligands will serve as improved starting scaffolds for therapeutic development.

### Species Specificity

AstraZeneca found that compound **B** only had off target activity against Moraxella catarrhalis.^28^ It showed no growth inhibition against 9 other bacterial species, along with Candida albicans and A549 human cells. While there is modest sequence similarity among the MurI enzymes, HpMurI is the only member known to form a head-to-head homodimer.^21^ Since the REAL compounds target the dimer interface like compound **B**, we anticipated a similar activity profile, as indicated by our preliminary specificity study.

### Future Directions

To enhance the activity of our lead compounds, several structural modifications can be considered. One hypothetical approach is to replace the intramolecular hydrogen bond with a new fused ring system, similar to compound **B**. This cyclization could potentially stabilize the bound state, increasing binding affinity to HpMurI. A more straightforward approach would focus on displacing additional water molecules from the dimer interface and expanding into the vacant space at the pocket entryways. Shown as yellow spheres, we observe several water molecules bound in the 2W4I crystal structure that could reasonably be replaced with substituents from slightly larger ligands (Figure 4d). Many of these sit at the pocket entrances, which could house bulky substituents if amenable to compound expansion (Figure 4e). By carefully building into these extended pockets through iterative optimization, we hope to improve potency while maintaining antibacterial activity.

## Conclusions

This study explores the synergy of giga sized drug-like chemical spaces with the challenges of drug discovery against Gram-negative pathogens. In doing so, we introduce a novel selective antibiotic class with significant potential for optimization. In order to advance these inhibitors toward clinical development, future investigations will need to more extensively evaluate bacterial selectivity, toxicity, in vivo PPB, and thoroughly explore Log *P* to balance potent MIC values with PPB. These steps are crucial for understanding our inhibitors’ therapeutic potential and improving their efficacy. If translatable to a clinical setting, we expect our antibiotics will be used in combination with standard of care therapies, particularly against drug-resistant strains. Since our inhibitors target peptidoglycan synthesis, it is conceivable that they may show synergistic activity when paired with other inhibitors of cell wall formation, such as β-lactams. As H. pylori infections are increasingly harder to treat through conventional means,^4^ the identification of a novel antibiotic holds great promise in combating the leading cause of gastric cancer, among other serious ailments.

Through the strategic use of Enamine’s REAL Space and insights from AstraZeneca’s inhibitors, we’ve identified a novel antibacterial class that showcases the pivotal role of structural diversity in combating Gram-negative bacteria. Notably, most compounds tested from REAL Space were new compositions of matter, which is often a prerequisite for further development upon these scaffolds. Our work not only demonstrates the successful implementation of REAL Space in antibiotic discovery and optimization, but also paves the way for future studies to expand upon the use of structure-based drug discovery to attain Gram-negative penetration. Overall, we present an innovative approach for the treatment of bacterial pathogens, providing a new avenue for addressing the global health challenge posed by antibiotic resistance.

## Experimental Section

### MOE Docking

All docking protocols were run in MOE2020. Decoy libraries were generated from the DUD-E server as previously described.^25^ Analogs of **1** were isolated from REAL Space by using infiniSee to search for compounds with >90% chemical similarity. Ligand libraries were washed at pH 7, scaled to reasonable bond lengths, and energy minimized. The crystal structure of compound **B** bound to the dimer interface (PDB: 2W4I) was prepared using the Quikprep utility in MOE. The docking site was defined by selecting the ligand atoms from 2W4I (VGA.E performed best) and docking against unselected atoms. For initial ROC tests, three poses were generated using Placement: Triangle matcher, Scoring function: London dG, Refinement: MMFF94x force field-based refinement, with various rescoring functions. Notably, the ligand in 2W4I primarily forms hydrogen bonds to water molecules surrounding the pocket (Figure S8a). Attempts to dock into the pocket without water drastically reduced AUC values. Because of this, we chose to keep all waters present in the crystal structure for docking. Data processing for ROC curves followed previous methods.^26^ Once GBVI/WSA was chosen as the top performing rescoring function, libraries were screened with two poses generated for each ligand entry. A threshold of *S* values below −10.0 was selected to determine compounds for visual inspection.

### HpMurI Expression and Induction

The gene encoding HpMurI (ATCC 700824) was inserted into pET15-b after the 6XHis tag. Separately, the chaperone GroEL/ES was inserted into pCH1. Both plasmids were transfected into E. coli BL21 DE3 pLysS cells and grown on Agar plates with 50 μg/mL ampicillin, 30 μg/mL chloramphenicol, and 100 μg/mL kanamycin. Single colonies were cultured overnight at 37 °C with shaking at 180 rpm in 50 mL of Terrific Broth (TB) divided into 5 tubes (10 mL each) supplemented with 50 μg/mL ampicillin, 30 μg/mL chloramphenicol, and 100 μg/mL kanamycin. The 5 × 10 mL starter cultures were combined and diluted into 4 flasks each containing 750 mL of TB medium with antibiotics. The cultures grew at 37 °C with shaking until the OD600 reached 0.8–1.0. Protein expression was induced upon addition of 0.1 mM IPTG and expressed for 16–18 h at 20 °C with shaking at 180 rpm. Cells were harvested by centrifugation at 5,000*g* at 4 °C for 20 min. Supernatant was discarded and cell pellets were either frozen for storage or resuspended in buffer for purification.

### HpMurI Protein Purification

Cell pellets of BL21 DE3 pLysS overexpressing HpMurI were resuspended in 2X ml per gram buffer A (100 mM Tris, 100 mM NaCl, 10 mM imidazole, 1 mM TCEP, pH 8.0) and supplemented with protease inhibitors. After vortexing to homogenize, 45 μL of 10 mg/mL DNaseI was added. The resuspension was passed through an Emulsiflex homogenizer at least three times at 10,000 psi. Insoluble matter was removed by centrifugation at 30,000*g* for 75 min at 4 °C and the supernatant was passed through a 0.22 μm filter. A HisTrap IMAC HP (GE Healthcare) cobalt resin column was equilibrated with buffer A (10 at 1 mL/min). Clarified lysate was then loaded onto the HisTrap column at 1 mL/min. HpMurI was then eluted by running a gradient of buffer A to buffer B (100 mM Tris, 100 mM NaCl, 500 mM imidazole, 1 mM TCEP, pH 8.0) over 50 mL.

Selected fractions were run on a SDS-PAGE Gel to determine purity and which fractions to pool. Pooled fractions were concentrated utilizing a 10,000 MWCO Amicon centrifugal filter device. After all selected fractions were combined and concentrated to less than 5 mL, the protein was diluted into SEC buffer (10 mM Tris, 100 mM NaCl, 1 mM TCEP, 10% glycerol, pH 8.0) in less than 2 mL. This was then loaded onto a pre-equilibrated HiLoad 16/200 Superdex column (GE Healthcare) and run with SEC buffer at 0.6 mL/min. Final fractions for pooling were determined via SDS-PAGE, and then concentrated to 5–7 mg/mL for storage at −80 °C.

### Enzyme-Coupled Activity Assay

Dose response curves were generated using a coupled assay to measure the conversion of d-glutamate to l-glutamate. Oxidation of l-glutamate to 2-oxoglutarate was catalyzed by l-glutamic acid dehydrogenase (LGDH), generating reduced β-nicotinamide adenine dinucleotide (NADH). NADH was then utilized by diaphorase to reduce iodonitrotetrazolium (INT), producing an absorption peak at 500 nm. Triplicate enzymatic reactions were conducted in clear flat-bottom 96-well microtiter plates at 25 °C, at a final volume of 100 μL. Plates were read for 500 nm absorbance using a Cary 300 UV–vis Spectrophotometer (Varian). The final assay composition was: 50 mM Tris at pH 8.0, 5 mM oxidized β-nicotinamide adenine dinucleotide, 37.5 units of LGDH, 2 mM adenosine diphosphate, 0.65 mM INT, 2 units diaphorase, 1 mM glutathione, and 1 μM HpMurI. The reaction was read for 1 h immediately following the addition of 50 μM d-glutamate. Reaction rates were calculated via linear regression of raw absorbance data. Rate values were fit to the log­(inhibitor) vs response (four parameters) function inside Graphpad-Prism 9.2.0.

### Differential Scanning Fluorimetry

Ligand effects on HpMurI thermal stability were carried out in triplicate using 96-well PCR plates. Purified HpMurI samples were diluted to 0.7 mg/mL in storage buffer supplemented with 10 mM d-glutamate. Test ligands in 100% DMSO were added to wells at 5% DMSO final, along with 6.25X SYPRO orange. Using a CFX Duet Real-Time PCR System, the temperature was increased at 0.5 °C per minute, while fluorescence emission was continuously monitored. Spectral data was processed using Melt Traceur to determine melting temperature Tm, and final plots were made in Graphpad-Prism 9.2.0.

### MIC Determination

The antibacterial activity HpMurI inhibitors was tested against H. pylori strains ATCC 43504 (metronidazole-resistant), ATCC 700684 (clarithromycin-resistant), and ATCC700392 (no resistance) following previously established protocols.^27^ H. pylori liquid cultures were grown in Brucella broth supplemented with 10% FBS and placed inside humidified microaerobic incubator (84% N2, 10% CO2, 6% O2) at 37 °C for 72 h. Prior to assay setup, each culture was diluted to a final optical density at 600 nm of 0.01 in culture media. 196 μL of diluted bacteria was added to all wells in the top row of a sterile 96-well flat-bottom microtiter plate, and 100 μL was added to all other wells. Next, 4 μL of 3.2 g/L compound stock (or 1.6 g/L in the case of metronidazole, clarithromycin, compound **A**) in 100% DMSO for each inhibitor were added to wells of the first row and carried over in a 2-fold serial dilution. DMSO, metronidazole, clarithromycin and compound **A** were included as controls. Each inhibitor was tested in a concentration range from 64 mg/L (or 32 mg/L for controls) to 0.5 mg/L (or 0.25 mg/L for controls) in triplicate. Plates were incubated under microaerobic conditions at 37 °C and measured for absorbance at 600 nm after 72 h. MIC values were defined as the lowest concentration of compound that showed no deviation from the antibiotic positive control (Clarithromycin for ATCC 43504 and metronidazole for ATCC 700684).

For MIC determination against E. coli MG1655, E. faecalis OG1RF, and B. subtilis PY79, we adapted this protocol for growth in a humidified incubator. Liquid cultures using Luria broth supplemented with 10% FBS were growth for 16 h at 37 °C with shaking. Cultures were then diluted to an optical density of 0.01 at 600 nm and plated as described previously. Compound activity was determined by measuring the absorbance at 600 nm after 48 h.

### d-Glutamate Rescue of H. pylori Growth

Liquid cultures of H. pylori strain ATCC 43504 were grown in Brucella broth supplemented with 10% FBS and placed inside humidified microaerobic incubator (84% N2, 10% CO2, 6% O2) at 37 °C for 72 h. After the OD600 reached 0.5, cultures were diluted into fresh Brucella broth containing 10% FBS, 50 mM NaCl, and 0.5 mM EDTA (pH 7.0) to enhance membrane permeability. Cells grew at a reduced replication rate for 72 h, or once the OD600 reached ∼0.8. Brucella broth with 10% FBS with the addition of 1 mM d-glutamate was brought to pH 7.0. Liquid H. pylori cultures were diluted to an OD600 of 0.01 in Brucella broth 10% FBS with and without additional d-glutamate. MICs were determined for compound **A**, **2**, metronidazole, and DMSO following the protocol discussed above. Cellular growth was normalized to the DMSO (for 100% growth) and metronidazole control (0% growth) to examine changes in antibacterial activity.

### Scanning Electron Microscopy

Strain ATCC 43504 was cultured in liquid media and exposed to compound **2** at concentrations of 2× to 1/8× MIC of (32 to 2 μg/mL), as well as DMSO. After 3 days of growth inside a humidified microaerobic incubator, 1 mL of each sample was centrifuged for 10 min at 14,000*g*. Culture media was aspirated, and 1 mL of 2.5% glutaraldehyde was added to each sample. Each sample was vortexed and left to incubate at 4 °C for 48 h. Samples were briefly spun at 5000*g* before being loaded onto 0.08 μm pore size filters wetted with 2 mL Dulbecco’s phosphate buffered saline. Sample filters were washed with 2 mL of 50%, 70%, 85%, 95% and 100% ethanol in increasing concentration, and subsequently dried with hexamethyldisilazane before being sputter-coated with gold. The prepared specimens were then examined using a Hitachi S-4000 scanning electron microscope operated at an accelerating voltage of 5.0 kV.

### Enamine REAL Compounds

All disclosed compounds were purchased commercially, with significant hits independently verified via NMR. All compounds are >95% pure by HPLC analysis. NMR spectra for primary compounds are listed in Figure S10. LC–MS traces provided by Enamine are listed in Figure S11.

## Supplementary Material















## Notes

Additional ROC curves, compound activity, ligand interaction maps, predicted physiochemical properties, molecular formula strings, NMR and HPLC purity analysis for compounds (PDF)
Compound_1_Docked (PDB)
Compound_2_Docked (PDB)
Compound_3_Docked (PDB)
Compound_4_Docked (PDB)
SMILES Spreadsheet (CSV)
NATx_Docked (PDB)

## Abbreviations

DSF: differential scanning fluorimetry
DUD-E: database of useful decoys enhanced
HpMurI: H. pylori glutamate racemase
MIC: minimum inhibitory concentration
MOE: molecular operating environment
MurI: glutamate racemase
PPB: plasma protein binding
REAL: readily accessible
ROC: receiver operating characteristic
SEM: scanning electron microscopy
