# Supplementary material for: Real Way to Target Gram-Negative Pathogens: Discovery of a Novel Helicobacter pylori Antibiotic Class
Source: J Med Chem. 2025 Mar 31;68(10):10128–38. doi: 10.1021/acs.jmedchem.5c00112 (PMC12105023; doi:10.1021/acs.jmedchem.5c00112)
Supplement: Supplementary file 1 [file jm5c00112_si_001.pdf]

Supporting Information

The REAL way to target Gram-negative pathogens: discovery of a novel *H. Pylori* antibiotic class

Jonah Pascal Propp,[1] Damien Oz Castor,[2] M. Ashley Spies[1,2]*

[1] *Division of Medicinal and Natural Products Chemistry, Department of Pharmaceutical Sciences and Experimental Therapeutics, The University of Iowa, Iowa City, Iowa 52242, United States of America*

[2] *Department of Biochemistry, Carver College of Medicine, The University of Iowa, Iowa City, Iowa 52242, United States of America*

Table of Contents

Figure S1. ROC curves for docking optimization

Figure S2. Compound 1 shows evidence of inhibition and binding

Figure S3. Similarity of REAL Ligands to NatX Hit

Figure S4. Activity Data for REAL Derivatives

Figure S5 Growth Inhibition of REAL Derivatives

Table S1. All Activity Data Collected on REAL Compounds

Figure S6. Partial Chemical Rescue of *H. pylori* with D-glutamate

Figure S7. REAL Inhibitors Show Species Specificity

Figure S8. ROC Curves of Alternate Docking Methods

Figure S9. 2D Ligand Interaction Map

Table S2. PPB Estimates

Figure S10. NMR Data for Primary Compounds

Figure S11. Mass Spectroscopy Data

Figure S12. PreADMET reports

ROC curves for docking optimization

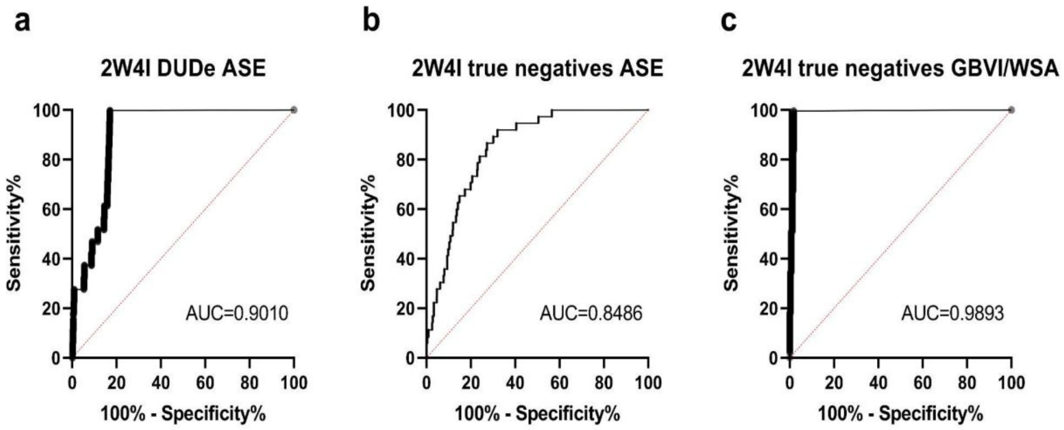

Figure S1: a, b, c, ROC curves for representative docking tests. The red diagonal represents true randomness (AUC=0.5), while a perfect docking protocol would show only a horizontal line at 100% sensitivity (AUC=1.0). **a,** ROC curve for docking using the ASE scoring function to evaluate 3 true positives against 300 decoys from the DUD-E server. **b,** ROC curve for the same protocol but with the inclusion of 65 true negatives. We observe an expected drop in the AUC with the addition of 65 ligands that scored favorably but were designated as true negatives. **c,** ROC for docking 3 true positives against the 65 true negatives that 'tricked' the ASE function.

Compound 1 shows evidence of inhibition and binding

a **b**

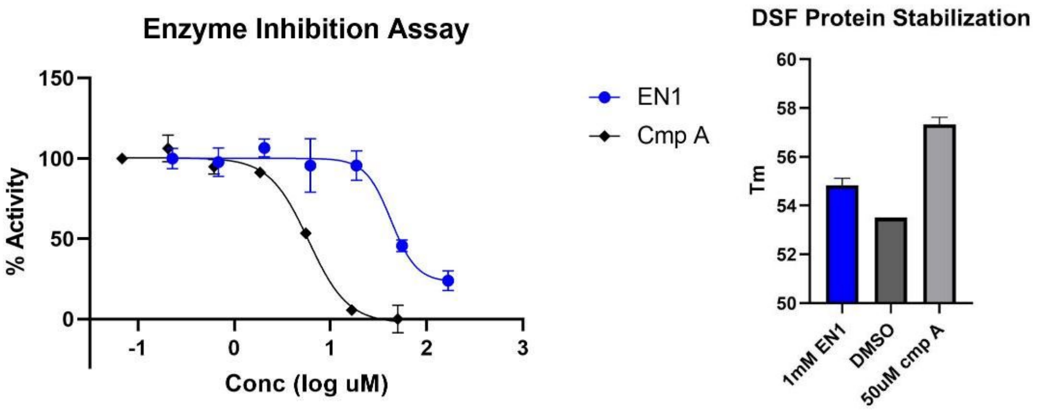

Figure S2: a, Coupled enzyme activity assay measuring turnover of D-glutamate to L-glutamate. **b,** HpMurI thermal stabilization measured by DSF

Similarity of REAL Ligands to NatX Hit

a **b**

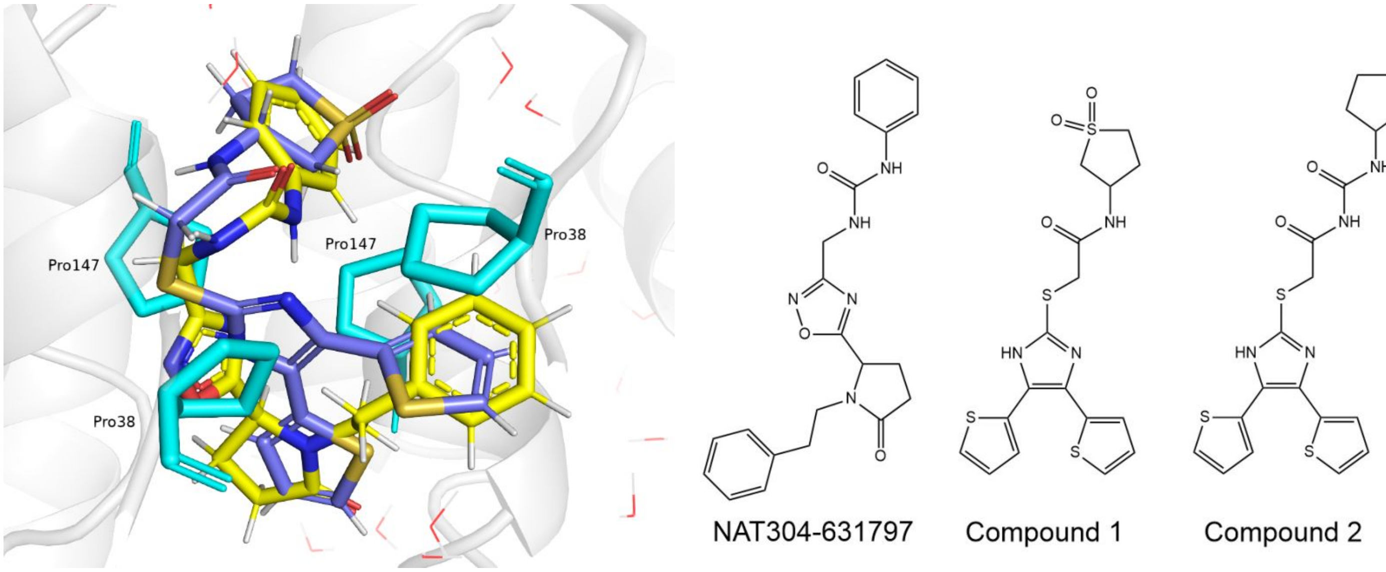

Figure S3: a, Overlay of docking poses for the initial Analyticon hit NAT304-631797 (Yellow), and compound 1(Purple) (PDBID: 2W4I used for modeling). We observe general similarities in terms of placement of rings and the linker/carbonyl. We also observe placement of the NATx hit phenyl ring in proximity to where we would expect for the proline groove, however it is shifted slightly outside of it. **b,** Lewis Structures for NAT304-631797, compound 1, and compound 2.

Activity Data for REAL Derivatives

a **b**

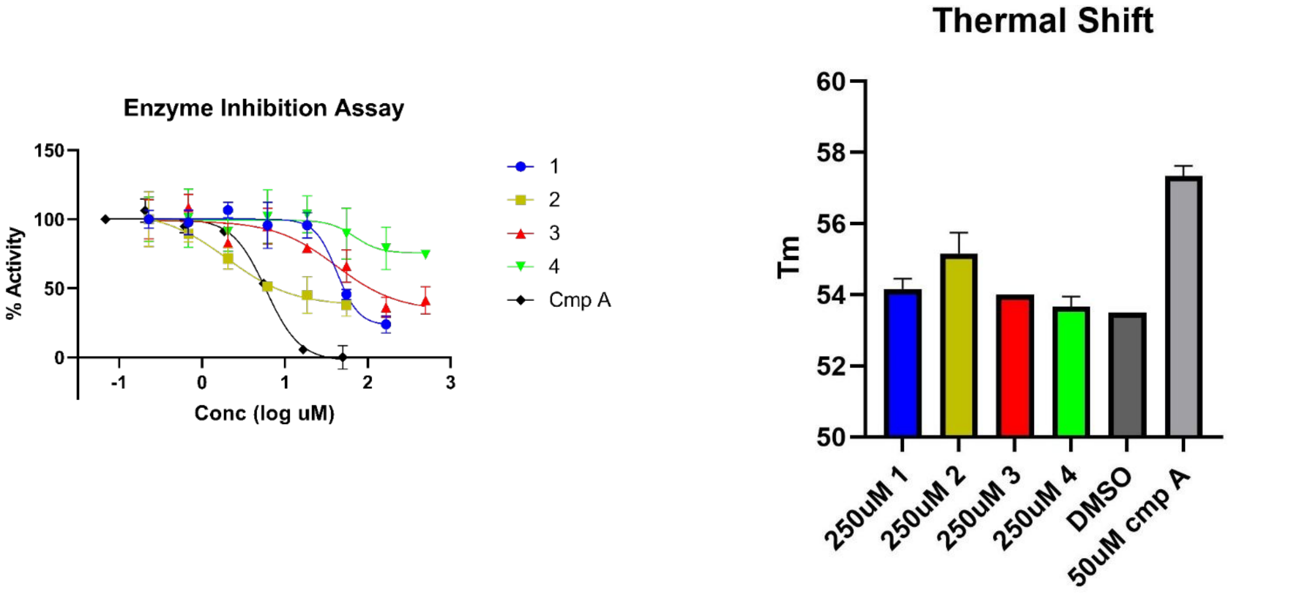

Figure S4: a, Coupled enzyme activity assay measuring turnover of D-glutamate to L-glutamate. **b,** HpMurI thermal stabilization measured by DSF.

Growth Inhibition of REAL Derivatives

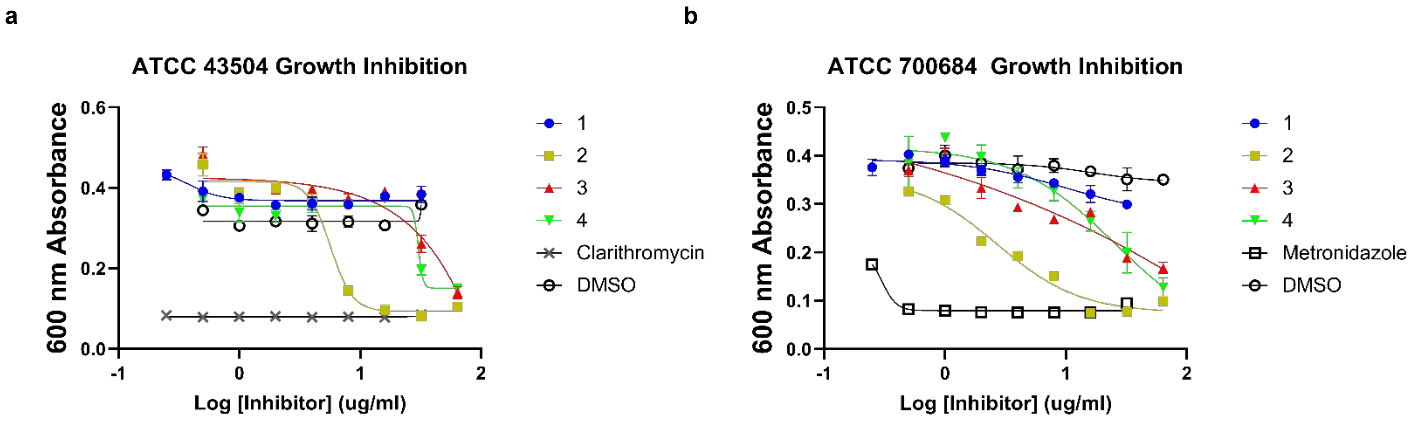

Figure S5: a-b, Antibacterial activity of **1, 2, 3, 4,** and controls against *H. pylori* strains ATCC 43504 (metronidazole resistant) and ATCC 700684 (clarithromycin resistant).[33] We observe that all second-generation inhibitors display improved growth inhibition compared to **1**. In particular, **2**, shows a potent ability to prevent *H. pylori* growth for both strains tested.

All Activity Data Collected on REAL Compounds

					Strain A	Strain A	Strain B	Strain B	Strain C	Strain C	
Cmp	Enamine ID	IC_{50}	% inhibition	Hill coefficient	MIC (ug/ml)	MIC_{50} (ug/ml)	MIC (ug/ml)	MIC_{50} (ug/ml)	MIC (ug/ml)	MIC_{50} (ug/ml)	LogP concesus
1	Z16940839	42.5	76.02	-3.274	>64	>64	>64	>64	>64	>64	2.95
2	Z16940993	2.002	63.67	-1.099	16	8	16	16	16	2	3.87
3	Z1090161132	41.7	66.47	-1.167	>64	64	64	64	>64	32	3.54
4	Z8853181462	69.3	26.74	-2.824	>64	32	64	64	>64	32	3.12
5	Z16940764	1.669	70.93	-1.392	32	16	32	32	N.D.	N.D.	3.76
6	Z16940308	16.78	87.28	-1.692	32	16	32	32	N.D.	N.D.	3.21
7	Z1157855181	15.65	68.39	-2.012	32	16	16	16	32	32	3.5
8	Z16940420	8.78	63.92	-1.783	16	8	16	16	32	32	3.5
9	Z16941695	10.72	75.48	-1.243	16	8	16	8	32	32	4.72
10	Z1116481184	3.888	59.67	-0.957	>64	16	N.D.	N.D.	>64	4	3.59
11	Z1533087985	12.3	47.41	-2.249	64	32	N.D.	N.D.	N.D.	N.D.	3.33
12	Z54730520	18.02	72.42	-1.673	>64	32	>64	>64	N.D.	N.D.	3.51
13	Z651249150	>250	N.D.	N.D.	N.D.	N.D.	N.D.	N.D.	N.D.	N.D.	N.D.
14	Z113385340	>250	N.D.	N.D.	N.D.	N.D.	N.D.	N.D.	N.D.	N.D.	N.D.
15	Z17621174	>250	N.D.	N.D.	N.D.	N.D.	N.D.	N.D.	N.D.	N.D.	N.D.
16	Z215370282	>250	N.D.	N.D.	N.D.	N.D.	N.D.	N.D.	N.D.	N.D.	N.D.
17	Z1397876329	>250	N.D.	N.D.	N.D.	N.D.	N.D.	N.D.	N.D.	N.D.	N.D.
18	Z83143470	>250	N.D.	N.D.	N.D.	N.D.	N.D.	N.D.	N.D.	N.D.	N.D.
19	Z30714510	>250	N.D.	N.D.	N.D.	N.D.	N.D.	N.D.	N.D.	N.D.	N.D.
20	Z18918800	>250	N.D.	N.D.	N.D.	N.D.	N.D.	N.D.	N.D.	N.D.	N.D.

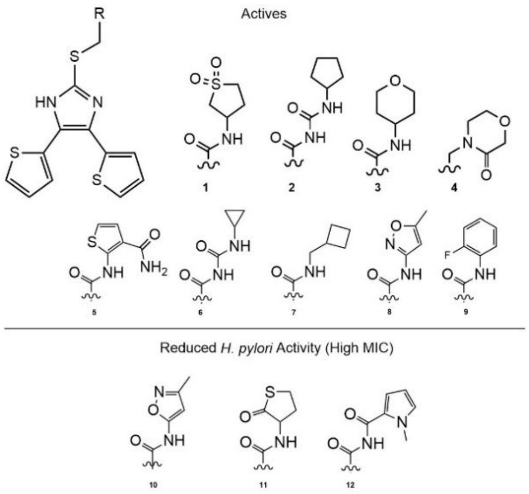

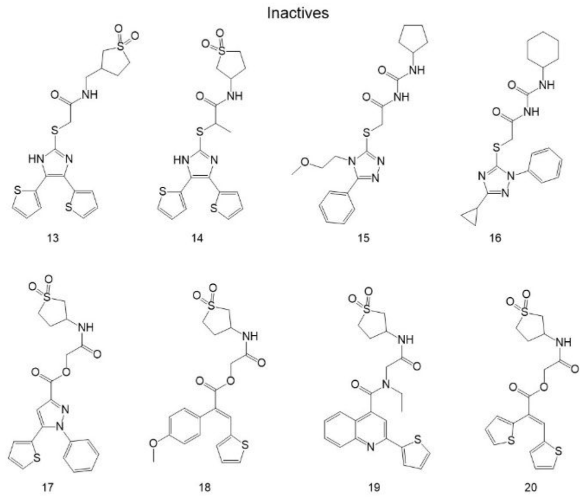

Table S1: Strain A= ATCC 45304, Strain B= ATCC 700392, Strain C= ATCC 700684

Partial Chemical Rescue of *H. pylori* with D-glutamate

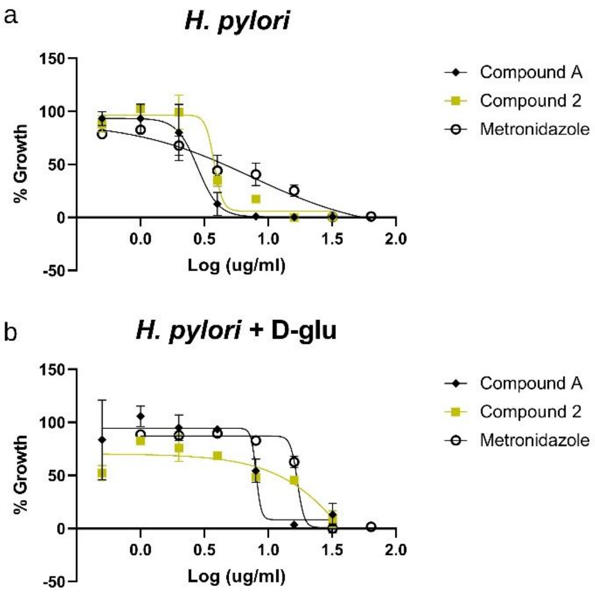

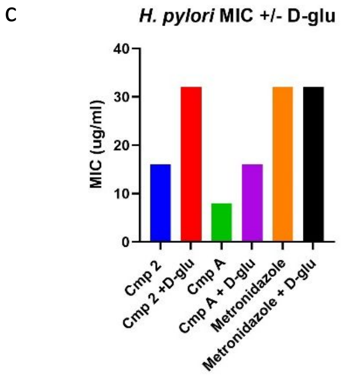

	No D-glu	With D-glu
Compound A	8 ug/ml	16 ug/ml
Compound 2	16 ug/ml	32 ug/ml
Metronidazole	32 ug/ml	32 ug/ml

Figure S6. Reduced MIC of HpMurI inhibitors in the presence of excess D-glutamate. (a) Growth curves for compounds A, **2**, and metronidazole under typical growth conditions. (b) Growth curves for compound A, **2**, and metronidazole in the presence of 1mM D-glutamate. A clear shift can be observed for A and **2** in the presence of 1mM D-glutamate in growth media. (c) MIC shifts plotted in bar graph format.

REAL Inhibitors Show Species Specificity

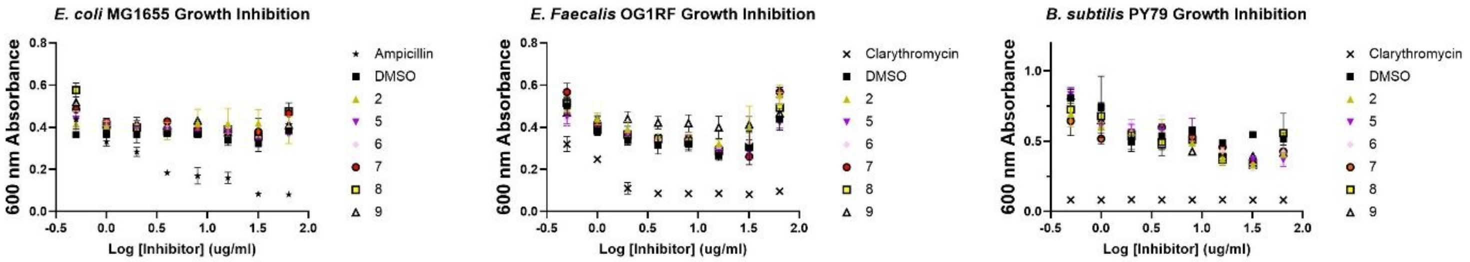

Figure S7: Activity of HpMurI inhibitors against three commensal gut bacteria strains. None of the REAL ligands showed evidence of growth inhibition up to 64 μg/ml.

ROC Curves of Alternate Docking Methods

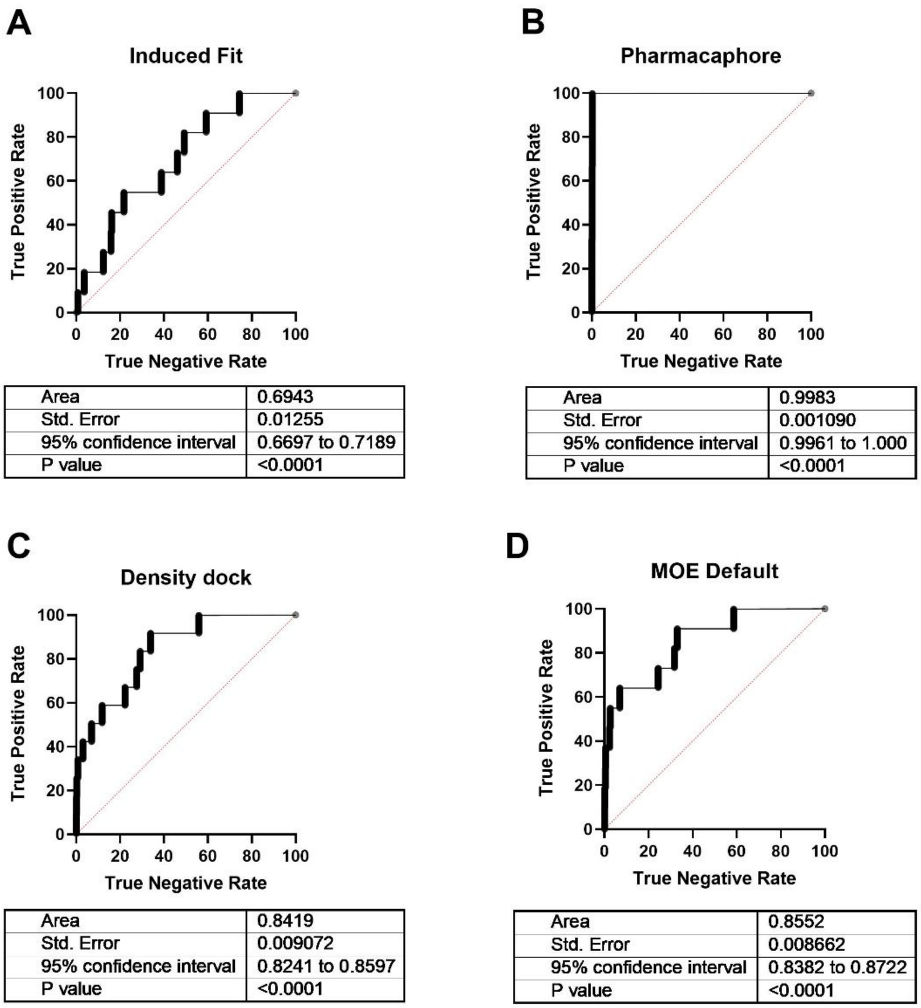

A — Induced Fit

Area	0.6943
Std. Error	0.01255
95% confidence interval	0.6697 to 0.7189
P value	<0.0001

B — Pharmacaphore

Area	0.9983
Std. Error	0.001090
95% confidence interval	0.9961 to 1.000
P value	<0.0001

C — Density dock

Area	0.8419
Std. Error	0.009072
95% confidence interval	0.8241 to 0.8597
P value	<0.0001

D — MOE Default

Area	0.8552
Std. Error	0.008662
95% confidence interval	0.8382 to 0.8722
P value	<0.0001

Figure S8: a-d, ROC curves for four additional docking protocol used in the optimization of our virtual screen.

2D Ligand Interaction Map

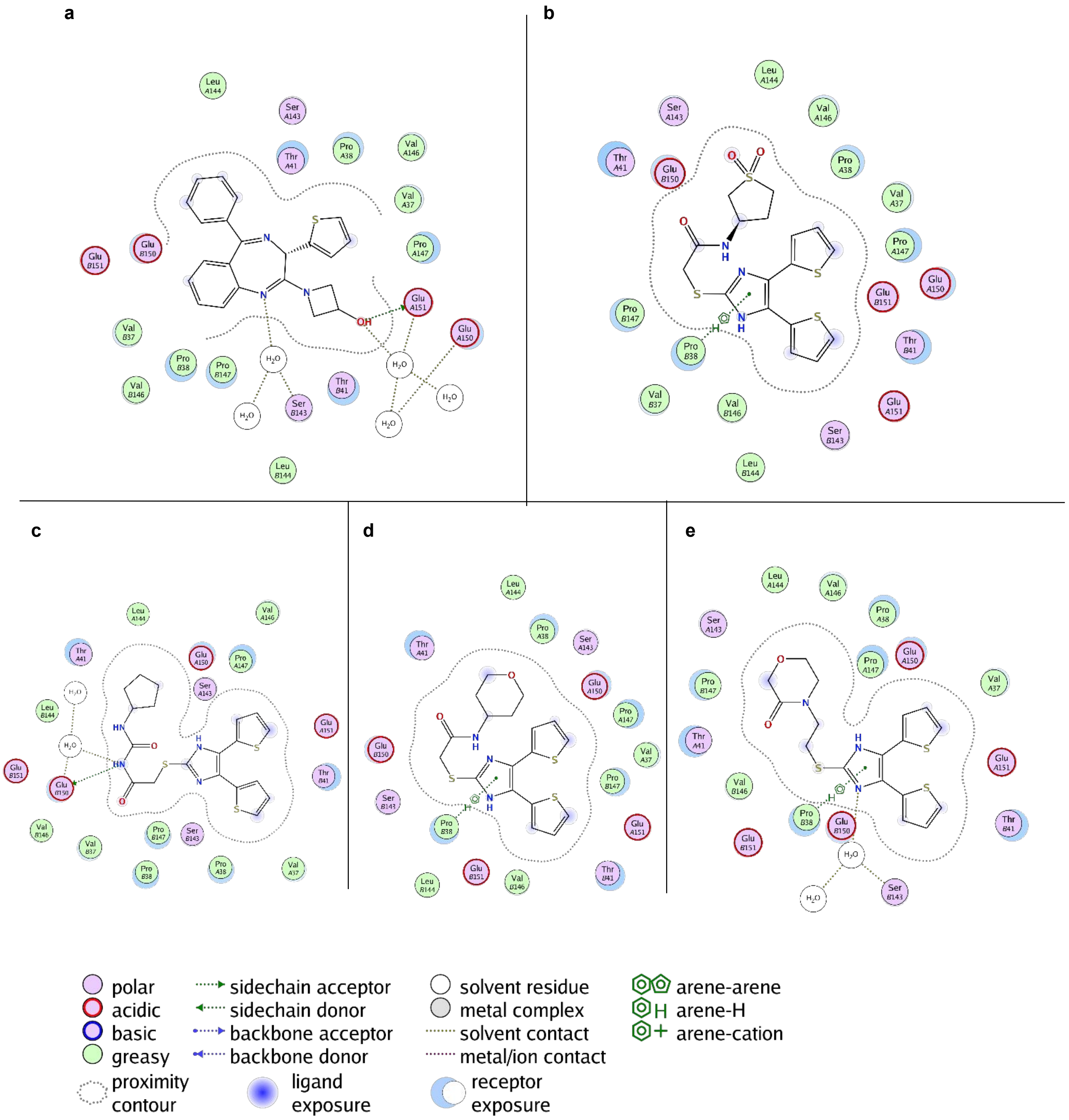

Figure S9: a-e, Ligand interactions maps for HpMurI inhibitors docked at the dimer interface in MOE (PDBID: 2W4I used for modeling). Interaction legend for maps is displayed at the bottom. **a,** Ligand interaction map for compound B. **b,** Ligand interaction map for compound 1. **c,** Ligand interaction map for compound 2. **d,** Ligand interaction map for compound 3. **e,** Ligand interaction map for compound 4.

PPB Estimates

	1	2	5	6	7	8	9	A	*B*
**PreADMET**	**100**	**85.67**	**100**	**86.89**	**94.72**	**88.14**	**98.13**	**90.91**	**95.27**
**ADMElab2.0**	**98.61**	**99.29**	**100.55**	**98.11**	**99.44**	**100.457**	**100.632**	**96.423**	**95.284**
**admetSAR**	**89.6**	**86.6**	**80.6**	**69.9**	**86.5**	**86.5**	**95.5**	**103.5**	**98.80**
**Consensus**	**96.0**	**90.5**	**93.7**	**84.9**	**93.5**	**91.7**	**98.1**	**97.0**	**96.4**

Table S2: PPB predictions for REAL compounds and controls using four different methods. PReADEMT, ADMElab2.0, and ademtSAR are all freely available software for *in vivo* property prediction. In general, we observe very poor agreement between methods. This is likely due to the limitation of training sets in prediction models, and our use of new chemical entities from REAL space. Notably, each method ranked compound **6** as either the most or second most favorable in terms of low PPB. To ease in interpreting these results, we report the 'consensus' PPB by averaging the four values.

Figure S10: NMR Data for Primary Compounds

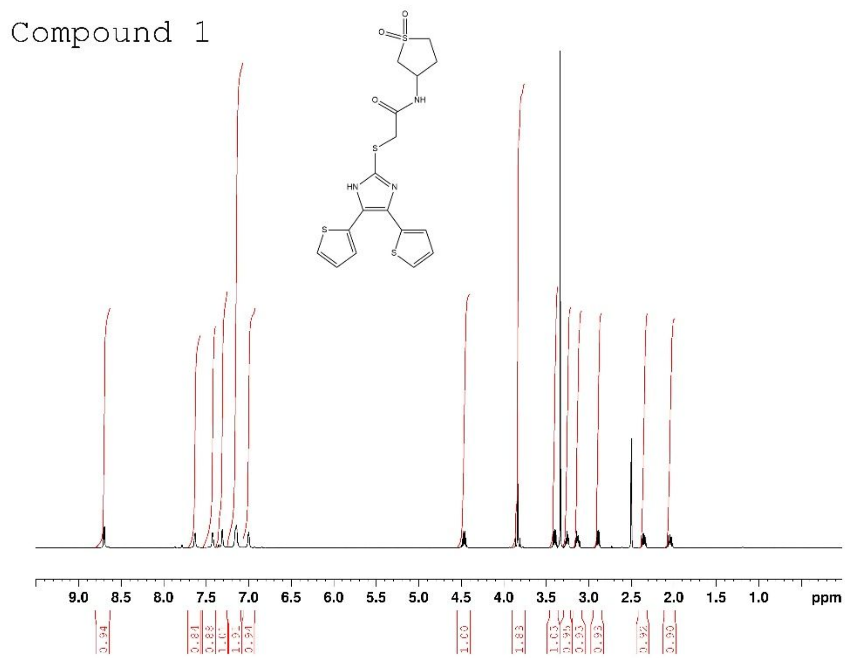

1H NMR (600 MHz, D6-DMSO): δ 8.71 (d, 1H), 7.63 (d, 1H), 7.42 (d, 1H), 7.31 (d, 1H), 7.16 (m, 2H), 7.00 (s, 1H), 4.46 (m, 1H), 3.83 (d, 2H), 3.39 (q, 1H), 3.25 (m, 1H), 3.13 (m, 1H), 2.89 (q, 1H), 2.35 (m, 1H), 2.04 (m, 1H).

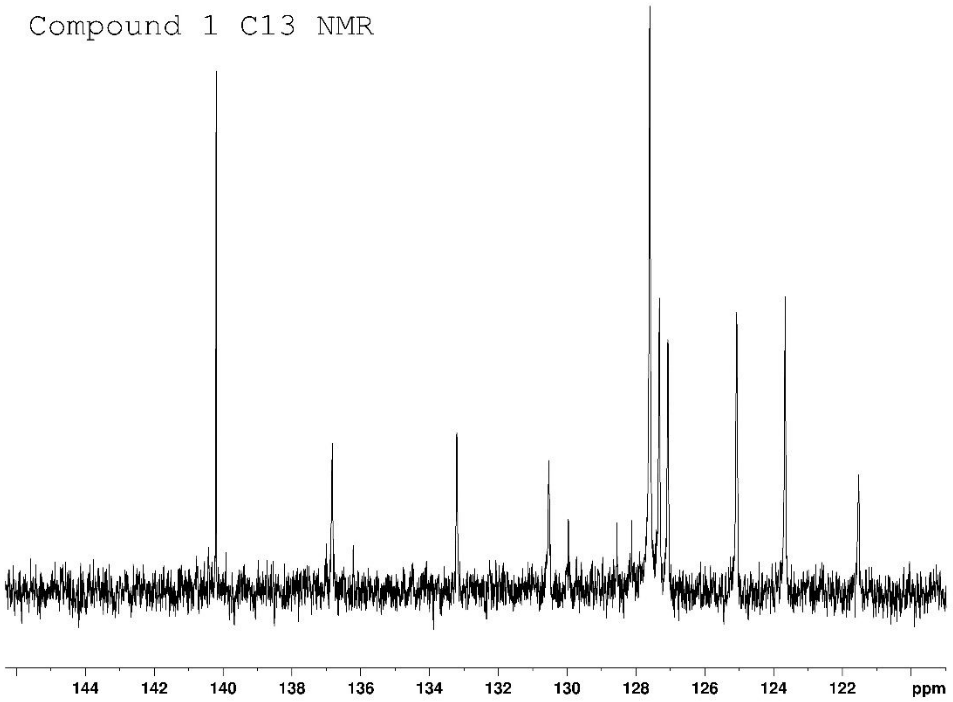

13C NMR (600 MHz, D6-DMSO): δ 167.5, 140.1, 136.8, 133.1, 130.5, 129.9, 127.5, 127.3, 127.0, 125.0, 123.6, 121.5, 55.1, 50.3, 46.0, 36.1, 28.2.

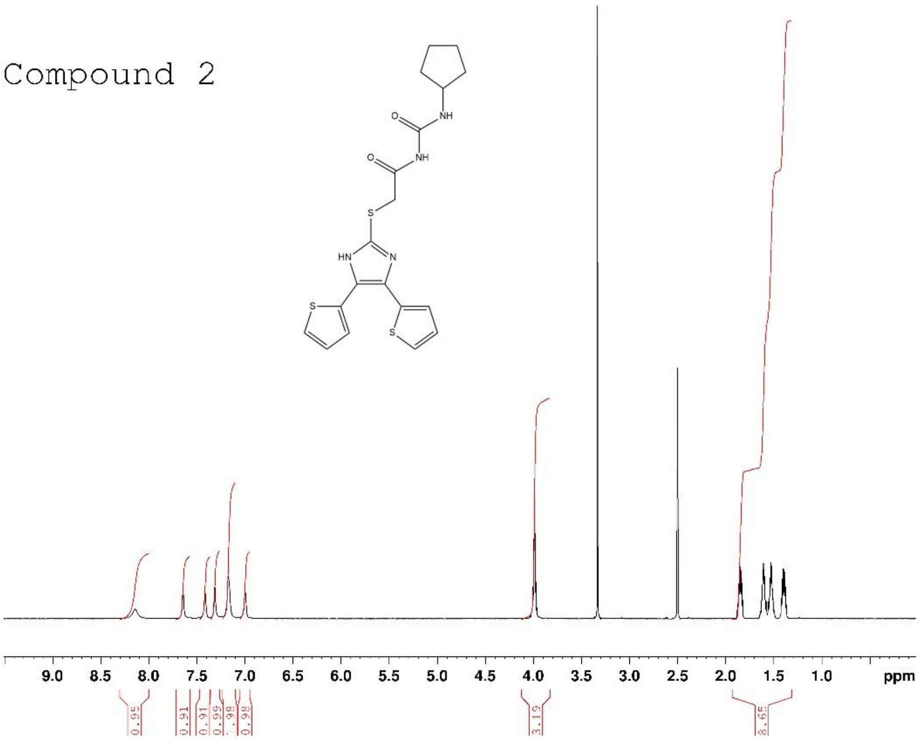

1H NMR (600 MHz, D6-DMSO): δ 8.14 (s, 1H), 7.65 (s, 1H), 7.41 (s, 1H), 7.31 (s, 1H), 7.17 (s, 2H), 6.99 (s, 1H), 3.98 (m, 3H), 1.84 (m, 2H), 1.60 (m, 2H), 1.52 (m, 2H), 1.35 (m, 2H).

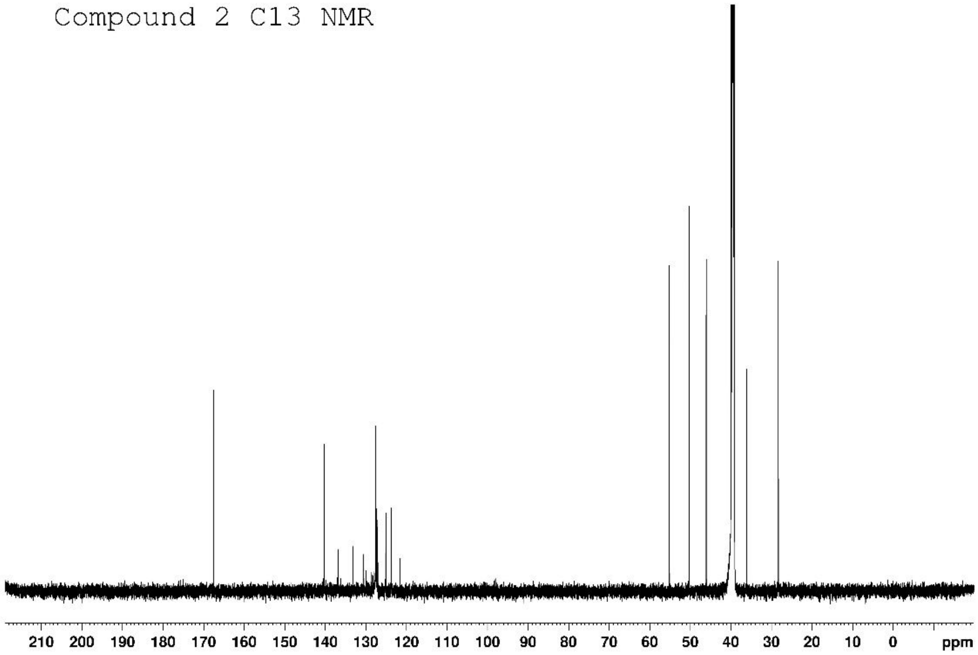

13C NMR (600 MHz, D6-DMSO): δ 170.4, 152.4, 139.9, 136.7 133.3, 130.4, 127.8, 127.6, 127.3, 127.1, 125.1, 123.8, 121.5, 50.8, 36.5, 32.5, 23.1

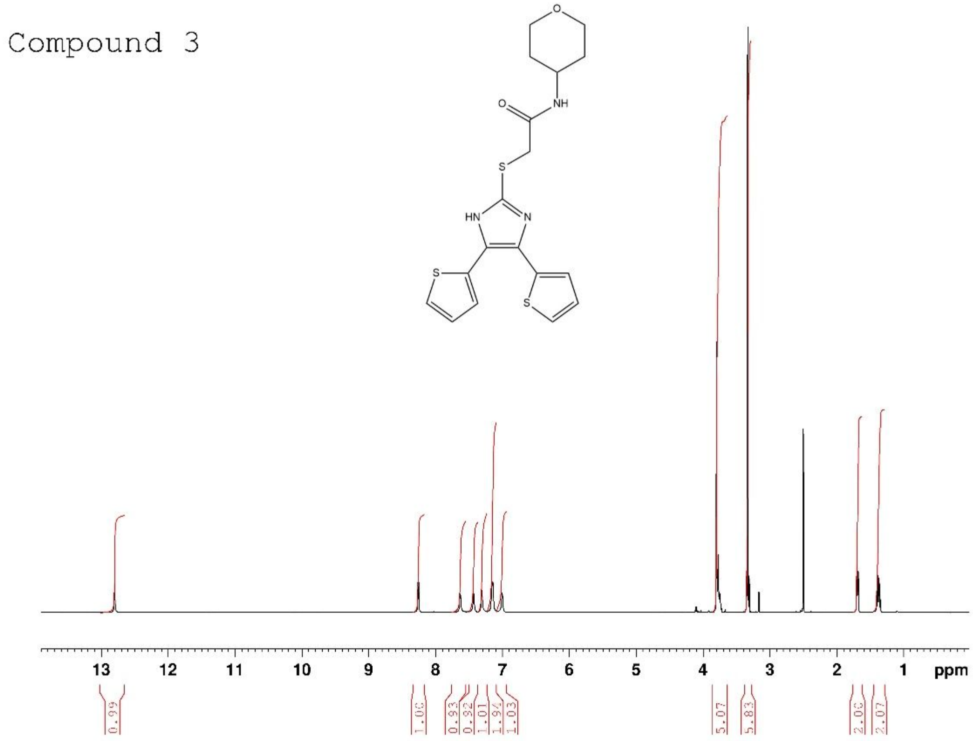

1H NMR (600 MHz, D6-DMSO): δ 12.81 (s, 1H), 8.25 (d, 1H), 7.63 (s, 1H), 7.43 (s, 1H), 7.31 (s, 1H), 7.14 (s, 2H), 7.00 (s, 1H), 3.78 (m, 4H), 3.32 (m, 2H overlapping with H2O contamination), 1.69 (d, 2H), 1.375 (m, 2H).

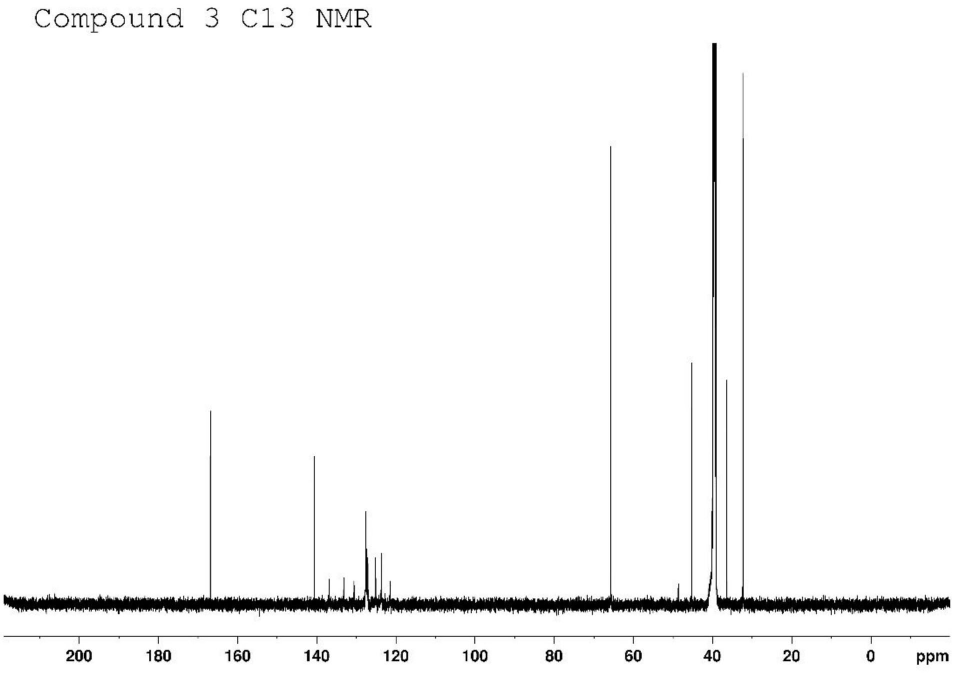

13C NMR (600 MHz, D6-DMSO): δ 166.8, 140.4, 136.8, 133.1, 130.5, 127.5, 127.3, 127.0, 125.0, 123.6, 121.4, 65.6, 48.5, 45.2, 36.3, 32.3.

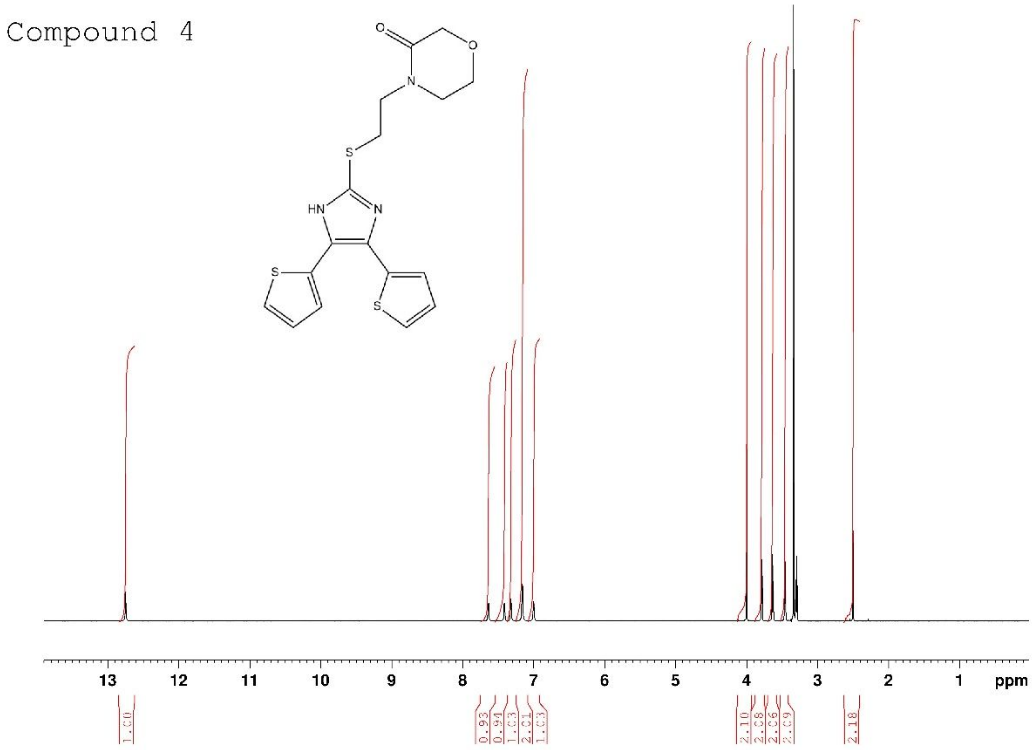

1H NMR (600 MHz, D6-DMSO): δ 12.76 (s, 1H), 7.63 (d, 1H), 7.41 (d, 1H), 7.32 (s, 1H), 7.16 (s, 2H), 7.00 (s, 1H), 7.00 (s, 1H), 3.99 (s, 2H), 3.78 (t, 2H), 3.63 (t, 2H), 3.45 (t, 2H), 3.29 (t, 2H), 2.50 (s, 2H).

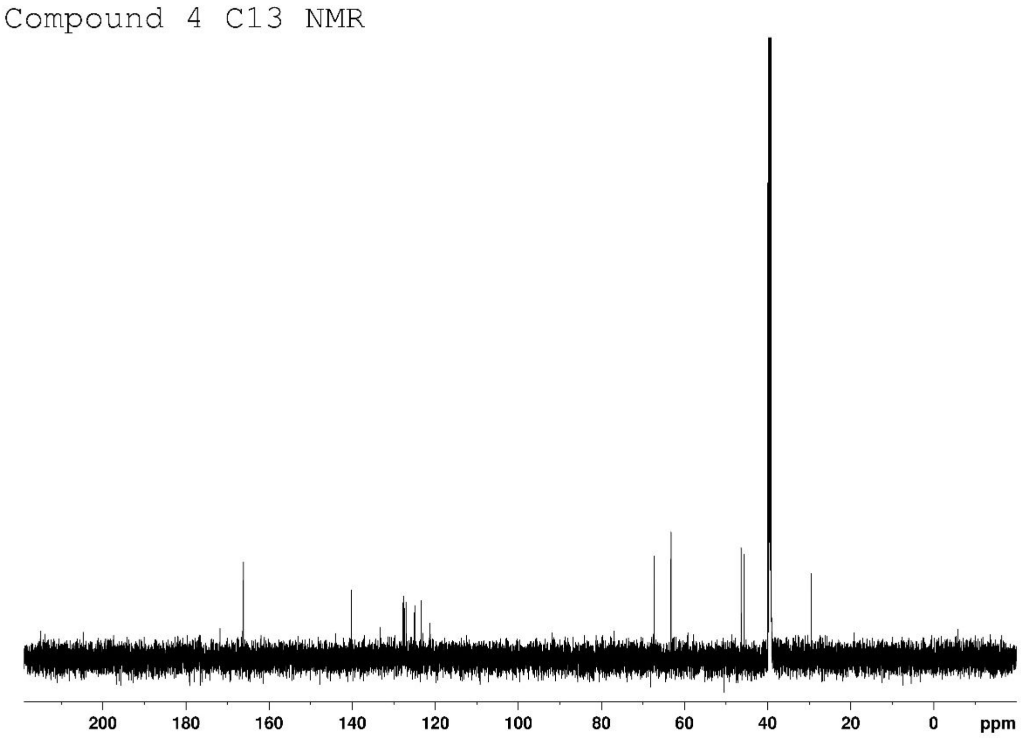

13C NMR (600 MHz, D6-DMSO): δ 166.1, 140.2, 127.6, 127.5, 127.3, 127.0, 124.9, 123.4, 67.2, 63.2, 46.3, 45.7, 29.4.

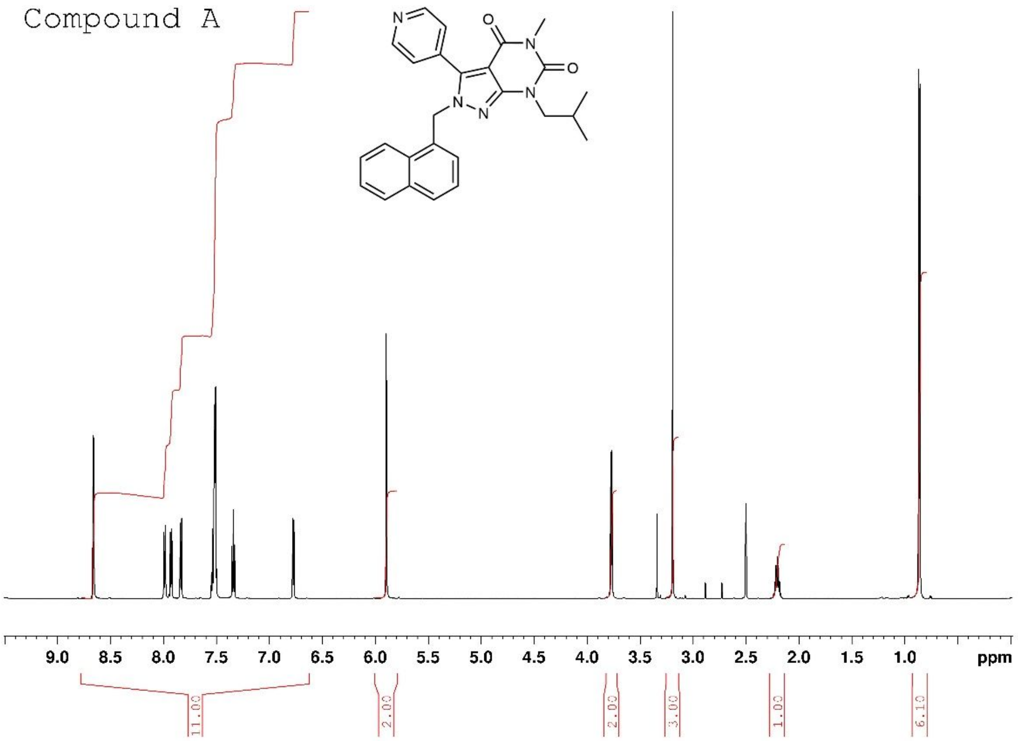

1H NMR (600 MHz, D6-DMSO): δ 8.66 (d, 2H), 7.99 (d, 1H), 7.93 (d, 1H), 7.83 (d, 1H), 7.51 (m, 4H), 7.33 (t, 1H), 7.00 (s, 1H), 6.77 (d, 1H), 5.89 (s, 2H), 3.77 (d, 2H), 3.19 (s, 3H), 2.20 (m, 1H), 0.86 (d, 6H).

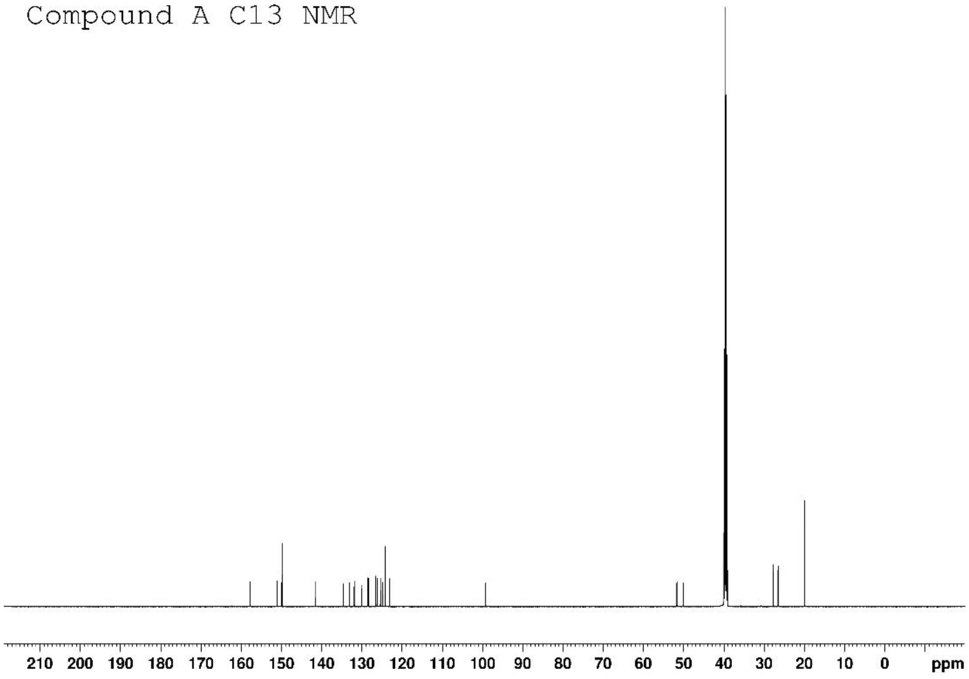

13C NMR (600 MHz, D6-DMSO): δ 157.7, 151.0, 149.9, 141.55 134.5, 133.0, 131.8, 129.9, 128.5, 128.3, 126.5, 126.1, 125.3, 124.7, 124.1, 123.0, 99.1, 51.6, 50.0, 27.6, 26.4, 19.8

Figure S11: Mass Spectroscopy Data

```
MaxPeak:   97.40%
Ret_Time:  1.026 min
```

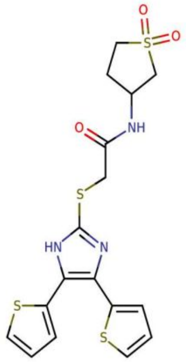

Mol Wt 439.6

Exact Mass 439.02

#	Time	Area%
1	0.972	2.60
2	1.026	97.40

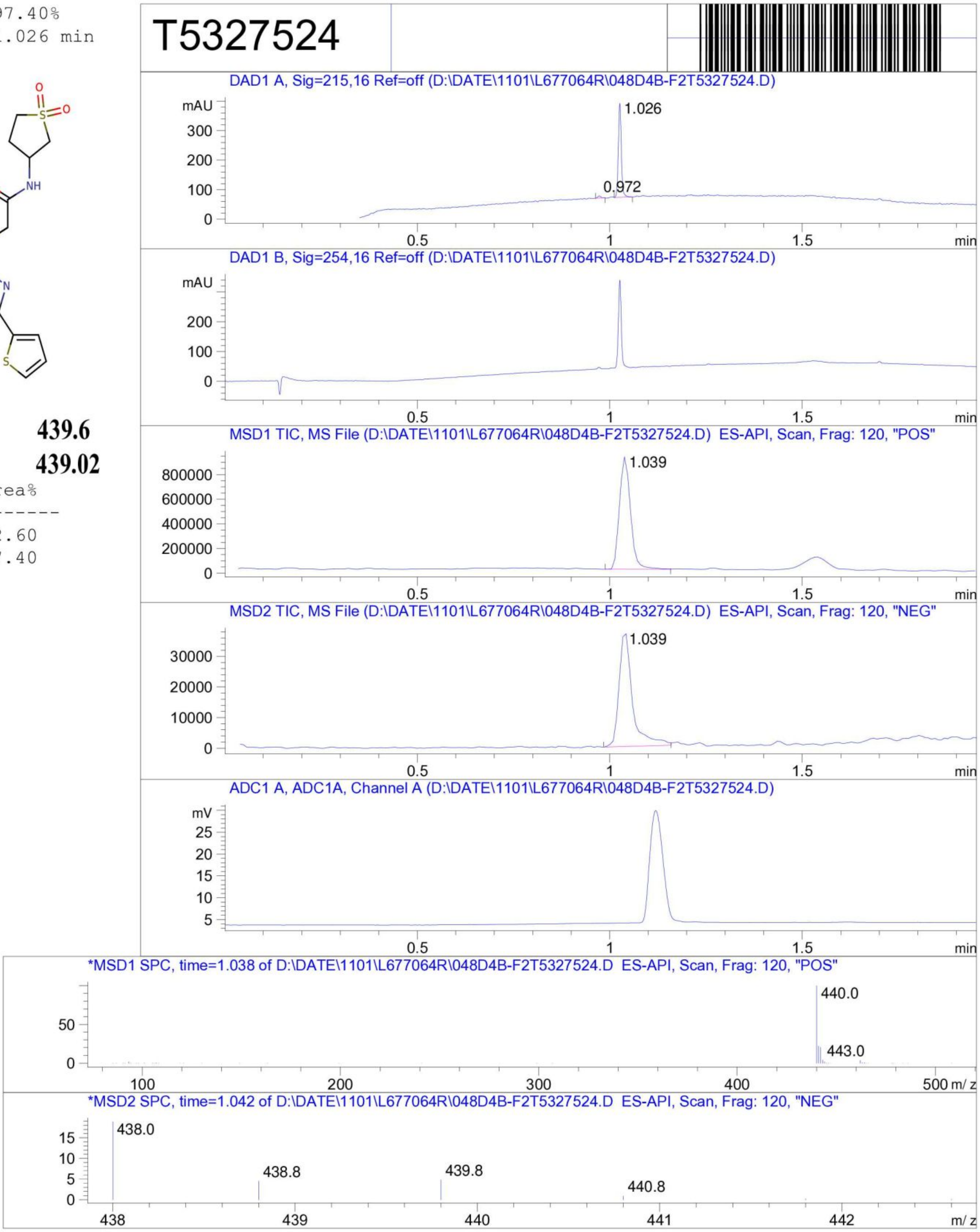

RT 1.039

RT 1.039

MaxPeak: 100.00%
Ret_Time: 1.371 min

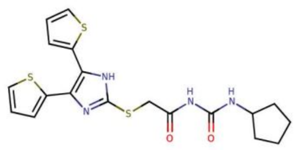

Mol Wt 432.58
Exact Mass 432.09

#	Time	Area%
1	1.371	100.00

BC904205$1

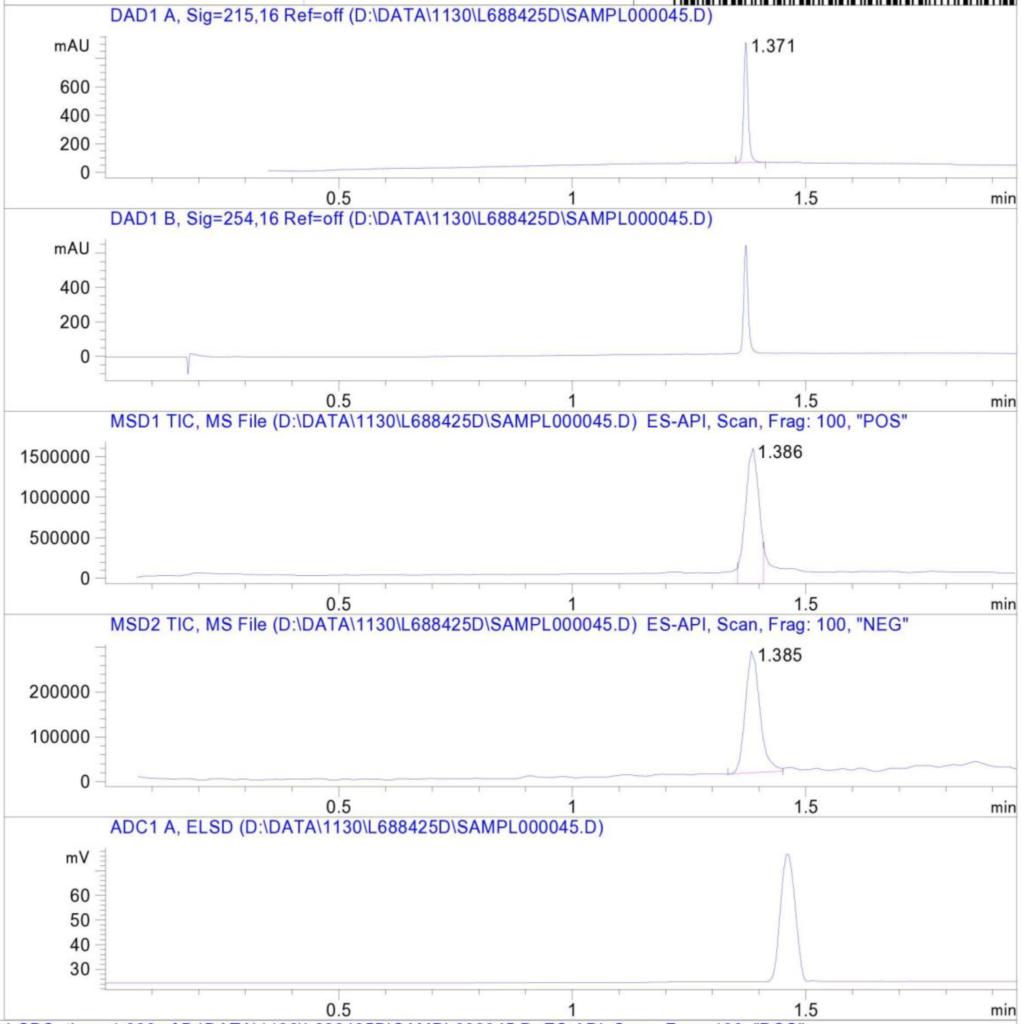

RT 1.386

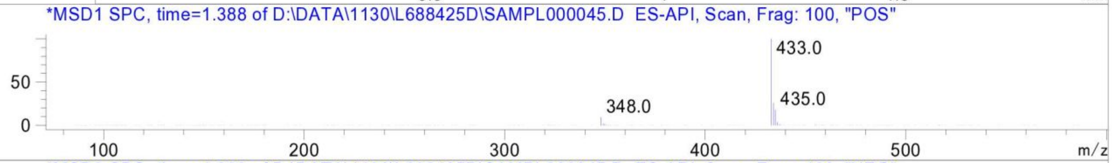

RT 1.385

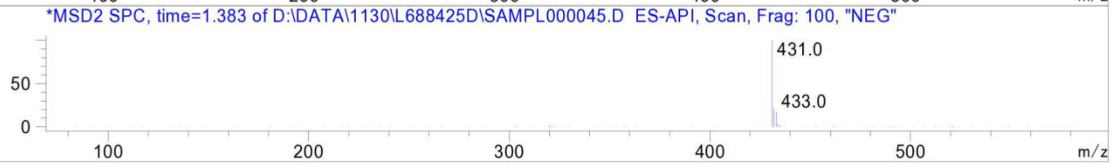

MaxPeak: 100.00%
Ret_Time: 1.204 min

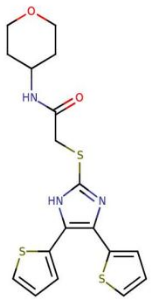

Mol Wt 405.56
Exact Mass 405.08

#	Time	Area%
1	1.204	100.00

BC904206$1

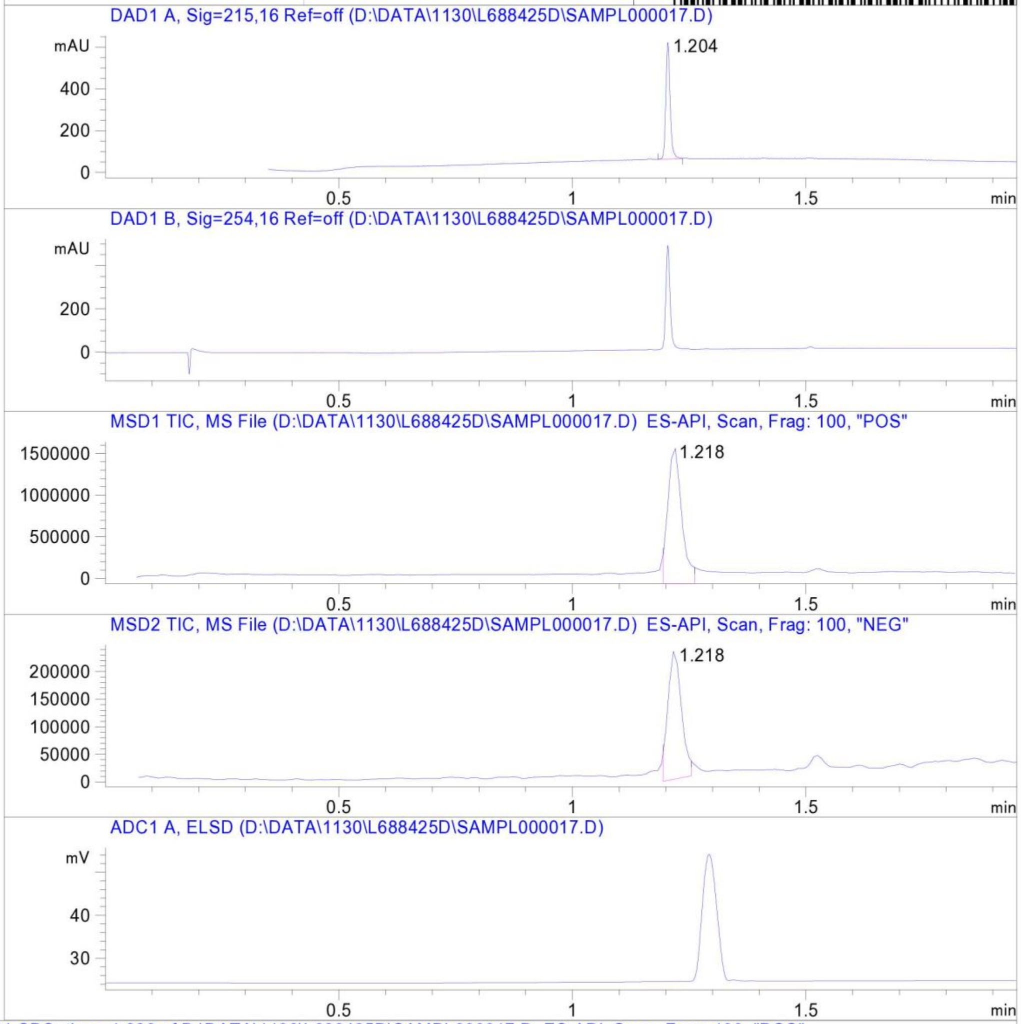

RT 1.218

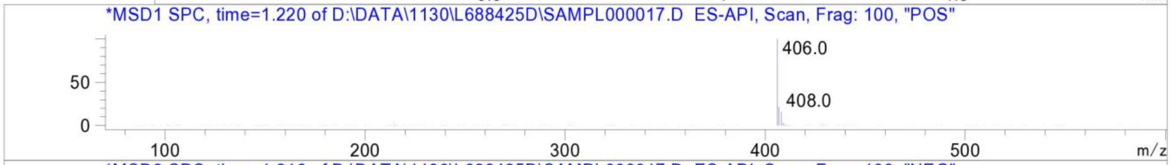

RT 1.218

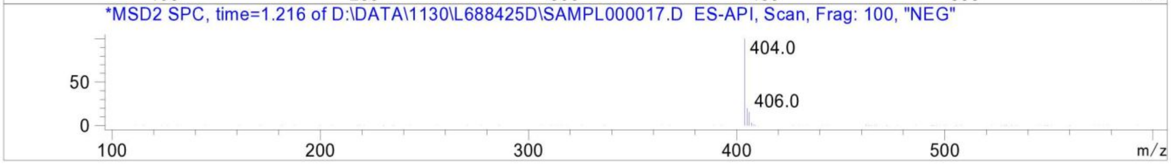

MaxPeak: 100.00%
Ret_Time: 1.175 min

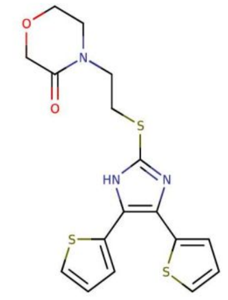

Mol Wt 391.53
Exact Mass 391.06

#	Time	Area%
1	1.175	100.00

BC904207$2

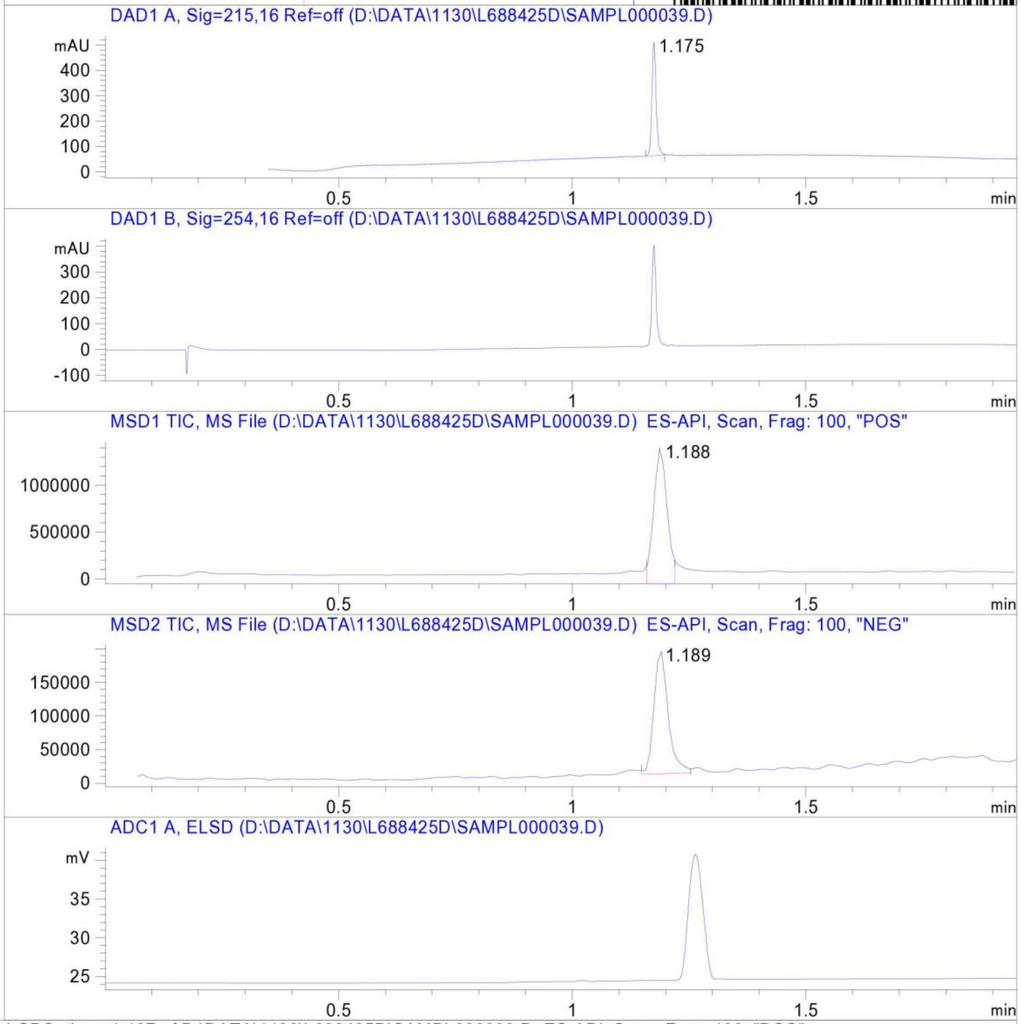

RT 1.188

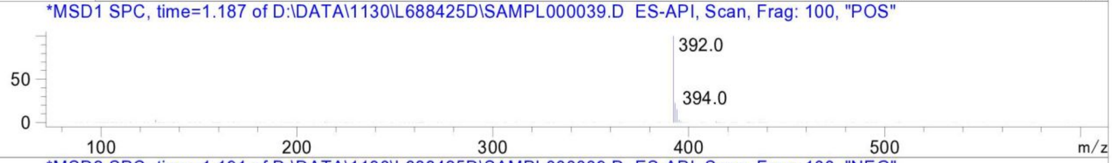

RT 1.189

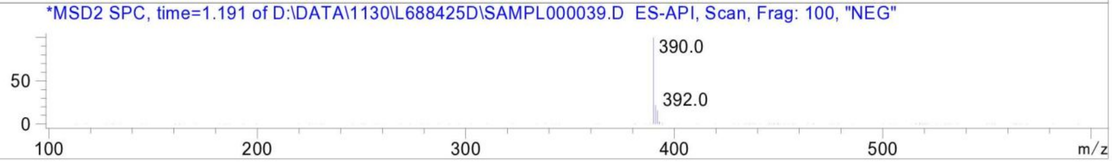

Figure S12: PreADMET reports

Molecule 1

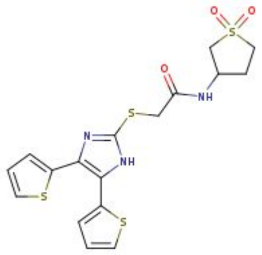

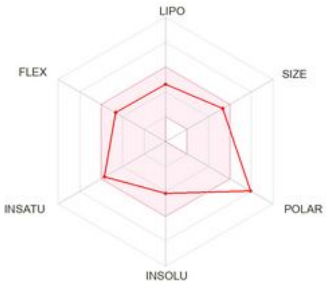

SMILES O=C(NC1CCS(=O)(=O)C1)CSc1[nH]c(c(n1)c1cccs1)c1cccs1

Physicochemical Properties	
Formula	C17H17N3O3S4
Molecular weight	439.60 g/mol
Num. heavy atoms	27
Num. arom. heavy atoms	15
Fraction Csp3	0.29
Num. rotatable bonds	7
Num. H-bond acceptors	4
Num. H-bond donors	2
Molar Refractivity	110.82
TPSA	182.08 Å²

Lipophilicity	
Log $P_{o/w}$ (iLOGP)	1.90
Log $P_{o/w}$ (XLOGP3)	2.58
Log $P_{o/w}$ (WLOGP)	4.34
Log $P_{o/w}$ (MLOGP)	0.94
Log $P_{o/w}$ (SILICOS-IT)	4.98
Consensus Log $P_{o/w}$	2.95

Water Solubility	
Log *S* (ESOL)	-4.14
Solubility	3.18e-02 mg/ml ; 7.24e-05 mol/l
Class	Moderately soluble
Log *S* (Ali)	-6.05
Solubility	3.90e-04 mg/ml ; 8.87e-07 mol/l
Class	Poorly soluble
Log *S* (SILICOS-IT)	-6.01
Solubility	4.27e-04 mg/ml ; 9.71e-07 mol/l
Class	Poorly soluble

Pharmacokinetics	
GI absorption	Low
BBB permeant	No
P-gp substrate	Yes
CYP1A2 inhibitor	Yes
CYP2C19 inhibitor	Yes
CYP2C9 inhibitor	Yes
CYP2D6 inhibitor	No
CYP3A4 inhibitor	Yes
Log K_p (skin permeation)	-7.15 cm/s

Druglikeness	
Lipinski	Yes; 0 violation
Ghose	Yes
Veber	No; 1 violation: TPSA>140
Egan	No; 1 violation: TPSA>131.6
Muegge	No; 1 violation: TPSA>150
Bioavailability Score	0.55

Medicinal Chemistry	
PAINS	0 alert
Brenk	0 alert
Leadlikeness	No; 1 violation: MW>350
Synthetic accessibility	3.96

Molecule 2

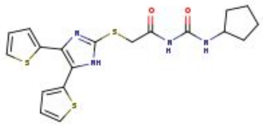

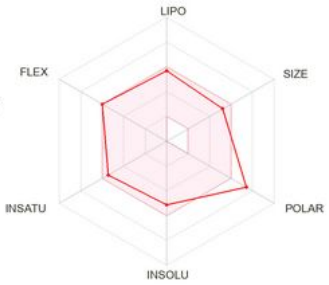

SMILES O=C(NC(=O)NC1CCCC1)CSc1[nH]c(c(n1)c1cccs1)c1cccs1

Physicochemical Properties	
Formula	C19H20N4O2S3
Molecular weight	432.58 g/mol
Num. heavy atoms	28
Num. arom. heavy atoms	15
Fraction Csp3	0.32
Num. rotatable bonds	9
Num. H-bond acceptors	3
Num. H-bond donors	3
Molar Refractivity	114.86
TPSA	168.66 Å²

Lipophilicity	
Log $P_{o/w}$ (iLOGP)	2.83
Log $P_{o/w}$ (XLOGP3)	4.44
Log $P_{o/w}$ (WLOGP)	4.73
Log $P_{o/w}$ (MLOGP)	1.89
Log $P_{o/w}$ (SILICOS-IT)	5.46
Consensus Log $P_{o/w}$	3.87

Water Solubility	
Log *S* (ESOL)	-5.12
Solubility	3.27e-03 mg/ml ; 7.56e-06 mol/l
Class	Moderately soluble
Log *S* (Ali)	-7.70
Solubility	8.62e-06 mg/ml ; 1.99e-08 mol/l
Class	Poorly soluble
Log *S* (SILICOS-IT)	-6.37
Solubility	1.83e-04 mg/ml ; 4.23e-07 mol/l
Class	Poorly soluble

Pharmacokinetics	
GI absorption	Low
BBB permeant	No
P-gp substrate	No
CYP1A2 inhibitor	Yes
CYP2C19 inhibitor	Yes
CYP2C9 inhibitor	Yes
CYP2D6 inhibitor	Yes
CYP3A4 inhibitor	Yes
Log K_p (skin permeation)	-5.79 cm/s

Druglikeness	
Lipinski	Yes; 0 violation
Ghose	Yes
Veber	No; 1 violation: TPSA>140
Egan	No; 1 violation: TPSA>131.6
Muegge	No; 1 violation: TPSA>150
Bioavailability Score	0.55

Medicinal Chemistry	
PAINS	0 alert
Brenk	0 alert
Leadlikeness	No; 3 violations: MW>350, Rotors>7, XLOGP3>3.5
Synthetic accessibility	3.53

Molecule 3

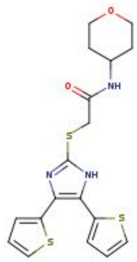

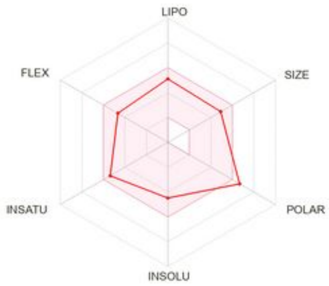

SMILES O=C(NC1CCOCC1)CSc1[nH]c(c(n1)c1cccs1)c1cccs1

Physicochemical Properties	
Formula	C18H19N3O2S3
Molecular weight	405.56 g/mol
Num. heavy atoms	26
Num. arom. heavy atoms	15
Fraction Csp3	0.33
Num. rotatable bonds	7
Num. H-bond acceptors	3
Num. H-bond donors	2
Molar Refractivity	107.75
TPSA	148.79 Å²

Lipophilicity	
Log $P_{o/w}$ (iLOGP)	2.76
Log $P_{o/w}$ (XLOGP3)	3.41
Log $P_{o/w}$ (WLOGP)	4.25
Log $P_{o/w}$ (MLOGP)	1.33
Log $P_{o/w}$ (SILICOS-IT)	5.92
Consensus Log $P_{o/w}$	3.54

Water Solubility	
Log *S* (ESOL)	-4.47
Solubility	1.38e-02 mg/ml ; 3.41e-05 mol/l
Class	Moderately soluble
Log *S* (Ali)	-6.21
Solubility	2.48e-04 mg/ml ; 6.10e-07 mol/l
Class	Poorly soluble
Log *S* (SILICOS-IT)	-6.14
Solubility	2.94e-04 mg/ml ; 7.26e-07 mol/l
Class	Poorly soluble

Pharmacokinetics	
GI absorption	Low
BBB permeant	No
P-gp substrate	Yes
CYP1A2 inhibitor	Yes
CYP2C19 inhibitor	Yes
CYP2C9 inhibitor	Yes
CYP2D6 inhibitor	Yes
CYP3A4 inhibitor	Yes
Log K_p (skin permeation)	-6.35 cm/s

Druglikeness	
Lipinski	Yes; 0 violation
Ghose	Yes
Veber	No; 1 violation: TPSA>140
Egan	No; 1 violation: TPSA>131.6
Muegge	Yes
Bioavailability Score	0.55

Medicinal Chemistry	
PAINS	0 alert
Brenk	0 alert
Leadlikeness	No; 1 violation: MW>350
Synthetic accessibility	3.38

Molecule 4

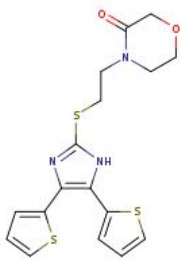

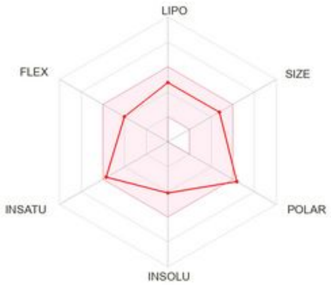

SMILES O=C1COCCN1CCSc1[nH]c(c(n1)c1cccs1)c1cccs1

Physicochemical Properties	
Formula	C17H17N3O2S3
Molecular weight	391.53 g/mol
Num. heavy atoms	25
Num. arom. heavy atoms	15
Fraction Csp3	0.29
Num. rotatable bonds	6
Num. H-bond acceptors	3
Num. H-bond donors	1
Molar Refractivity	106.95
TPSA	140.00 Å^2

Lipophilicity	
Log $P_{o/w}$ (iLOGP)	2.49
Log $P_{o/w}$ (XLOGP3)	2.82
Log $P_{o/w}$ (WLOGP)	3.44
Log $P_{o/w}$ (MLOGP)	1.09
Log $P_{o/w}$ (SILICOS-IT)	5.75
Consensus Log $P_{o/w}$	3.12

Water Solubility	
Log *S* (ESOL)	-4.09
Solubility	3.17e-02 mg/ml ; 8.09e-05 mol/l
Class	Moderately soluble
Log *S* (Ali)	-5.42
Solubility	1.50e-03 mg/ml ; 3.82e-06 mol/l
Class	Moderately soluble
Log *S* (SILICOS-IT)	-5.73
Solubility	7.26e-04 mg/ml ; 1.85e-06 mol/l
Class	Moderately soluble

Pharmacokinetics	
GI absorption	Low
BBB permeant	No
P-gp substrate	Yes
CYP1A2 inhibitor	Yes
CYP2C19 inhibitor	Yes
CYP2C9 inhibitor	Yes
CYP2D6 inhibitor	Yes
CYP3A4 inhibitor	Yes
Log K_p (skin permeation)	-6.69 cm/s

Druglikeness	
Lipinski	Yes; 0 violation
Ghose	Yes
Veber	Yes
Egan	No; 1 violation: TPSA>131.6
Muegge	Yes
Bioavailability Score	0.55

Medicinal Chemistry	
PAINS	0 alert
Brenk	0 alert
Leadlikeness	No; 1 violation: MW>350
Synthetic accessibility	3.32

Molecule 1

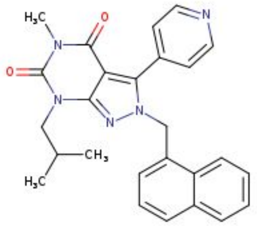

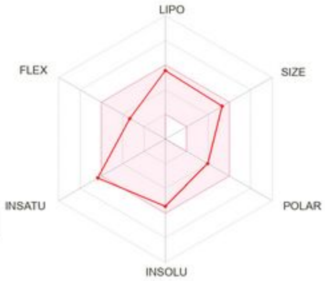

SMILES CC(Cn1c2nn(c(c2c(=O)n(c1=O)C)c1ccncc1)Cc1cccc2c1cccc2)C

Physicochemical Properties	
Formula	C26H25N5O2
Molecular weight	439.51 g/mol
Num. heavy atoms	33
Num. arom. heavy atoms	25
Fraction Csp3	0.23
Num. rotatable bonds	5
Num. H-bond acceptors	4
Num. H-bond donors	0
Molar Refractivity	131.68
TPSA	74.71 Å^2

Lipophilicity	
Log $P_{o/w}$ (iLOGP)	3.43
Log $P_{o/w}$ (XLOGP3)	4.22
Log $P_{o/w}$ (WLOGP)	3.82
Log $P_{o/w}$ (MLOGP)	3.26
Log $P_{o/w}$ (SILICOS-IT)	3.91
Consensus Log $P_{o/w}$	3.73

Water Solubility	
Log *S* (ESOL)	-5.45
Solubility	1.54e-03 mg/ml ; 3.51e-06 mol/l
Class	Moderately soluble
Log *S* (Ali)	-5.50
Solubility	1.39e-03 mg/ml ; 3.17e-06 mol/l
Class	Moderately soluble
Log *S* (SILICOS-IT)	-7.75
Solubility	7.87e-06 mg/ml ; 1.79e-08 mol/l
Class	Poorly soluble

Pharmacokinetics	
GI absorption	High
BBB permeant	Yes
P-gp substrate	Yes
CYP1A2 inhibitor	Yes
CYP2C19 inhibitor	Yes
CYP2C9 inhibitor	Yes
CYP2D6 inhibitor	No
CYP3A4 inhibitor	Yes
Log K_p (skin permeation)	-5.98 cm/s

Druglikeness	
Lipinski	Yes; 0 violation
Ghose	No; 1 violation: MR>130
Veber	Yes
Egan	Yes
Muegge	Yes
Bioavailability Score	0.55

Medicinal Chemistry	
PAINS	0 alert
Brenk	0 alert
Leadlikeness	No; 2 violations: MW>350, XLOGP3>3.5
Synthetic accessibility	3.58

Molecule 2

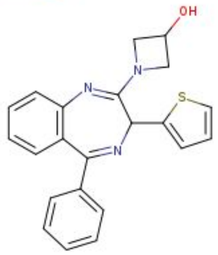

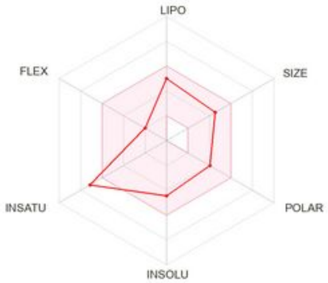

SMILES OC1CN(C1)C1=Nc2ccccc2C(=NC1c1cccs1)c1ccccc1

Physicochemical Properties	
Formula	C22H19N3OS
Molecular weight	373.47 g/mol
Num. heavy atoms	27
Num. arom. heavy atoms	17
Fraction Csp3	0.18
Num. rotatable bonds	3
Num. H-bond acceptors	3
Num. H-bond donors	1
Molar Refractivity	120.78
TPSA	76.43 Å²

Lipophilicity	
Log $P_{o/w}$ (iLOGP)	2.94
Log $P_{o/w}$ (XLOGP3)	3.24
Log $P_{o/w}$ (WLOGP)	2.58
Log $P_{o/w}$ (MLOGP)	2.91
Log $P_{o/w}$ (SILICOS-IT)	5.43
Consensus Log $P_{o/w}$	3.42

Water Solubility	
Log *S* (ESOL)	-4.46
Solubility	1.28e-02 mg/ml ; 3.43e-05 mol/l
Class	Moderately soluble
Log *S* (Ali)	-4.52
Solubility	1.13e-02 mg/ml ; 3.03e-05 mol/l
Class	Moderately soluble
Log *S* (SILICOS-IT)	-6.67
Solubility	7.97e-05 mg/ml ; 2.13e-07 mol/l
Class	Poorly soluble

Pharmacokinetics	
GI absorption	High
BBB permeant	Yes
P-gp substrate	No
CYP1A2 inhibitor	No
CYP2C19 inhibitor	Yes
CYP2C9 inhibitor	Yes
CYP2D6 inhibitor	Yes
CYP3A4 inhibitor	Yes
Log K_p (skin permeation)	-6.28 cm/s

Druglikeness	
Lipinski	Yes; 0 violation
Ghose	Yes
Veber	Yes
Egan	Yes
Muegge	Yes
Bioavailability Score	0.55

Medicinal Chemistry	
PAINS	0 alert
Brenk	0 alert
Leadlikeness	No; 1 violation: MW>350
Synthetic accessibility	4.77
